# Differential Effects of Attention-, Compassion-, and Socio-Cognitively Based Mental Practices on Self-Reports of Mindfulness and Compassion

**DOI:** 10.1007/s12671-017-0716-z

**Published:** 2017-04-06

**Authors:** Lea K. Hildebrandt, Cade McCall, Tania Singer

**Affiliations:** 1 Max Planck Institute for Human Cognitive and Brain Sciences, Stephanstraße 1a, 04103 Leipzig, Germany; 2 Department of Psychology, University of Würzburg, Würzburg, Germany; 3 Department of Psychology, University of York, York, UK

**Keywords:** Mindfulness, Compassion, Self-compassion, Questionnaires, Longitudinal design

## Abstract

Research on the effects of mindfulness- and compassion-based interventions is flourishing along with self-report scales to assess facets of these broad concepts. However, debates remain as to which mental practices are most appropriate to develop the attentional, cognitive, and socio-affective facets of mindfulness and compassion. One crucial question is whether present-moment, attention-focused mindfulness practices are sufficient to induce a cascade of changes across the different proposed facets of mindfulness, including nonjudgmental acceptance, as well as compassion or whether explicit socio-affective training is required. Here, we address these questions in the context of a 9-month longitudinal study (the ReSource Project) by examining the differential effects of three different 3-month mental training modules on subscales of mindfulness and compassion questionnaires. The “Presence” module, which aimed at cultivating present-moment-focused attention and body awareness, led to increases in the observing, nonreacting, and presence subscales, but not to increases in acceptance or nonjudging. These latter facets benefitted from specific cultivation through the socio-cognitive “Perspective” module and socio-affective, compassion-based “Affect” module, respectively. These modules also led to further increases in scores on the subscales affected by the Presence module. Moreover, scores on the compassion scales were uniquely influenced by the Affect module. Thus, whereas a present-moment attention-focused training, as implemented in many mindfulness-based programs, was indeed able to increase attentional facets of mindfulness, only socio-cognitive and compassion-based practices led to broad changes in ethical-motivational qualities like a nonjudgmental attitude, compassion, and self-compassion.

**Electronic supplementary material:**

The online version of this article (doi:10.1007/s12671-017-0716-z) contains supplementary material, which is available to authorized users.

## Introduction

The interest in mindfulness and compassion-based intervention programs such as Mindfulness-Based Stress Reduction (MBSR; Kabat-Zinn 1982), Mindfulness-Based Cognitive Therapy (MBCT; Segal et al. 2002), or Compassion Cultivation Training (CCT; Jazaieri et al. 2013a, b) is growing as these types of interventions have been shown to have a variety of beneficial effects, such as decreasing depression, anxiety, or chronic pain (e.g., Baer 2003; Galante et al. 2014; Grossman et al. 2004). The concept of mindfulness in particular has received a great deal of attention in the last decade of psychological and neuroscientific research. However, descriptions of mindfulness vary considerably (c.f. Bishop et al. 2004; Chiesa 2013; Grossman and Van Dam 2011; Hanley et al. 2016; Rapgay and Bystrisky 2009; Vago and Silbersweig 2012) and the term is nowadays used interchangeably to describe states, traits, psychological functions, and cognitive processes, as well as different types of meditation practices and entire intervention programs (Vago and Silbersweig 2012). While there is a general agreement that mindfulness crucially involves attention to, and awareness of, the present moment (Brown and Ryan 2003, 2004; but see Dreyfus 2011; Grossman and Van Dam 2011; Grossman 2008), controversy remains regarding the relationship between those capacities and concepts such as acceptance and nonjudgmental awareness. Moreover, it is unclear whether present-moment, attention-focused mindfulness practices are sufficient to elicit a cascade of changes including acceptance, nonjudgmental awareness, and compassion, or whether explicit practices are needed to bring about these socio-affective and motivational qualities. Here, we therefore tested the specific effect of different types of mental practices on different subscales of widely used mindfulness and compassion-related self-report questionnaires.

In the Buddhist literature, the Pali and Sanskrit words for mindfulness can be translated as “to remember” or “to keep in mind” (Dreyfus 2011), which represents, according to Dreyfus, a sort of sustained attention to the object in mind. Rapgay and Bystrisky (2009) define classical mindfulness as a perceptual process of “bare experience” (p. 158). An often-cited, modern definition of mindfulness is “paying attention in a particular way, on purpose, in the present moment, and nonjudgmentally” (p. 4, Kabat-Zinn 1994). Based on this definition, Bishop et al. (2004) have proposed that mindfulness consists of two facets: (1) self-regulation of attention to the present moment and (2) an openness to and acceptance of experience. This subdivision parallels the differentiation into presence and acceptance found in the short form of the Freiburg Mindfulness Inventory (FMI; Kohls et al. 2009; Sauer et al. 2011), a questionnaire based on the definitions of mindfulness of expert meditators. Some research groups (e.g., Shapiro et al. 2006; see also Vago and Silbersweig 2012) have subdivided mindfulness into even more than two facets. This plurality of conceptualizations has resulted in a number of different self-report mindfulness questionnaires (see Baer et al. 2006; Bergomi et al. 2013). To reduce these scales to their common essence, Baer et al. (2006) combined items of several mindfulness scales in a factor analysis and identified five separate factors: *observing* inner experiences, *acting with awareness*, *describing* inner experiences, *nonreacting* to inner experiences, and *nonjudging* of experience.

While general agreement exists that mindfulness crucially involves attention to, and awareness of, the present moment (Brown and Ryan 2003, 2004; but see Dreyfus 2011; Grossman and Van Dam 2011; Grossman 2008), controversy remains regarding whether acceptance and nonjudgmental awareness are part of the core concept. Some classically oriented accounts of mindfulness, such as those presented by Rapgay and Bystrisky (2009), Dreyfus (2011), or Bodhi (2011), specifically exclude ethical-motivational processes like nonjudgmental acceptance. Rapgay and Bystrisky (2009) argue that classical mindfulness is a purely perceptual process that excludes any meta-cognitive evaluations or preconceptions such as acceptance. Furthermore, they, as well as Dreyfus (2011), argue that mindfulness is not nonjudgmental, as it depends on judgment to differentiate right from wrong states of mind. Some have further argued that nonjudgment and acceptance represent a warm, caring attitude that is better represented as compassion or self-compassion (Hofmann et al. 2011; Shapiro et al. 2006).

Compassion denotes a feeling of concern and care in response to another person’s suffering, coupled with a motivation to alleviate the suffering and improve the other’s well-being (Goetz et al. 2010; Singer and Klimecki 2014), whereas self-compassion is this feeling of care directed at oneself (Neff 2003). Conceptually, mindfulness and compassion are interrelated constructs and integral parts of the Buddhist practice that can be difficult to isolate (Chiesa 2013; Grossman and Van Dam 2011, Kabat-Zinn 2003). According to some Buddhist traditions, mindfulness, as part of the Noble Eightfold Path, needs to be embedded in a compassionate, ethical stance oriented towards nonharming and a friendly presence (Kabat-Zinn 2003). Thus, especially the second part of the definition of mindfulness, nonjudgmental acceptance, indicates that compassion and mindfulness are intrinsically linked: compassion is found in mindfulness as well as mindfulness in compassion (Germer and Barnhofer 2017). This is reflected in the research on mindfulness practices. Mindfulness training programs have been not only associated with increases in the various facets of mindfulness (e.g., Baer et al. 2008) but have further been associated with increased self-compassion and compassion (Birnie et al. 2010; Gu et al. 2015; Keng et al. 2012; Neff and Dahm 2015; Salzberg 2011). One might conclude from these findings that present-moment-focused practices, including drawing one’s attention to the breath or the body scan (two core meditations practices in many mindfulness-based interventions; Kabat-Zinn 1982), have broad-reaching effects by triggering a “mindfulness cascade” which go beyond affecting attention and awareness to increasing acceptance, nonjudgment, compassion, and self-compassion (e.g., Brown and Ryan 2004; Grossman 2008).

One problem with this interpretation, however, is that mindfulness-based intervention programs, especially the popular MBSR (Kabat-Zinn 1982), often contain a wide variety of different meditation and mental training practices (Hofmann et al. 2011). These may implicitly include (self-)compassion-based or other psycho-educative or therapeutic features (Eberth and Sedlmeier 2012; Hanley et al. 2016; Neff and Dahm 2015) that further incorporate affect-related themes (Neff and Dahm 2015; Rapgay and Bystrisky 2009). In addition, mindfulness-based interventions often include meditation practices such as observation of thoughts or open awareness practices. These latter practices may be more likely to train ethical-motivational aspects than the pure presence- and attention-based exercises. As a consequence, it is unclear whether such mindfulness-based intervention programs cultivate a core mindfulness which, in turn, triggers the “mindfulness cascade,” or rather if the different practices involved in these trainings each uniquely influence specific facets of mindfulness, compassion, or self-compassion. According to the latter, alternative view, ethical-motivational qualities—including nonjudgmental attitudes, acceptance, and compassion—require specific, targeted cultivation through compassion and acceptance-based mental training practices (Desbordes et al. 2015; Neff and Dahm 2015; Vago and Silbersweig 2012). These ethical-motivational qualities may, in fact, be the critical mechanisms through which mindfulness leads to improved mental health and well-being (e.g., Birnie et al. 2010; Desbordes et al. 2015; Gu et al. 2015; Keng et al. 2012; Woodruff et al. 2014). Therefore, the question remains whether these different types of contemplative practices lead to differential improvements on more basic, attentional versus socio-affective and ethical-motivational qualities.

**Fig. 1:**
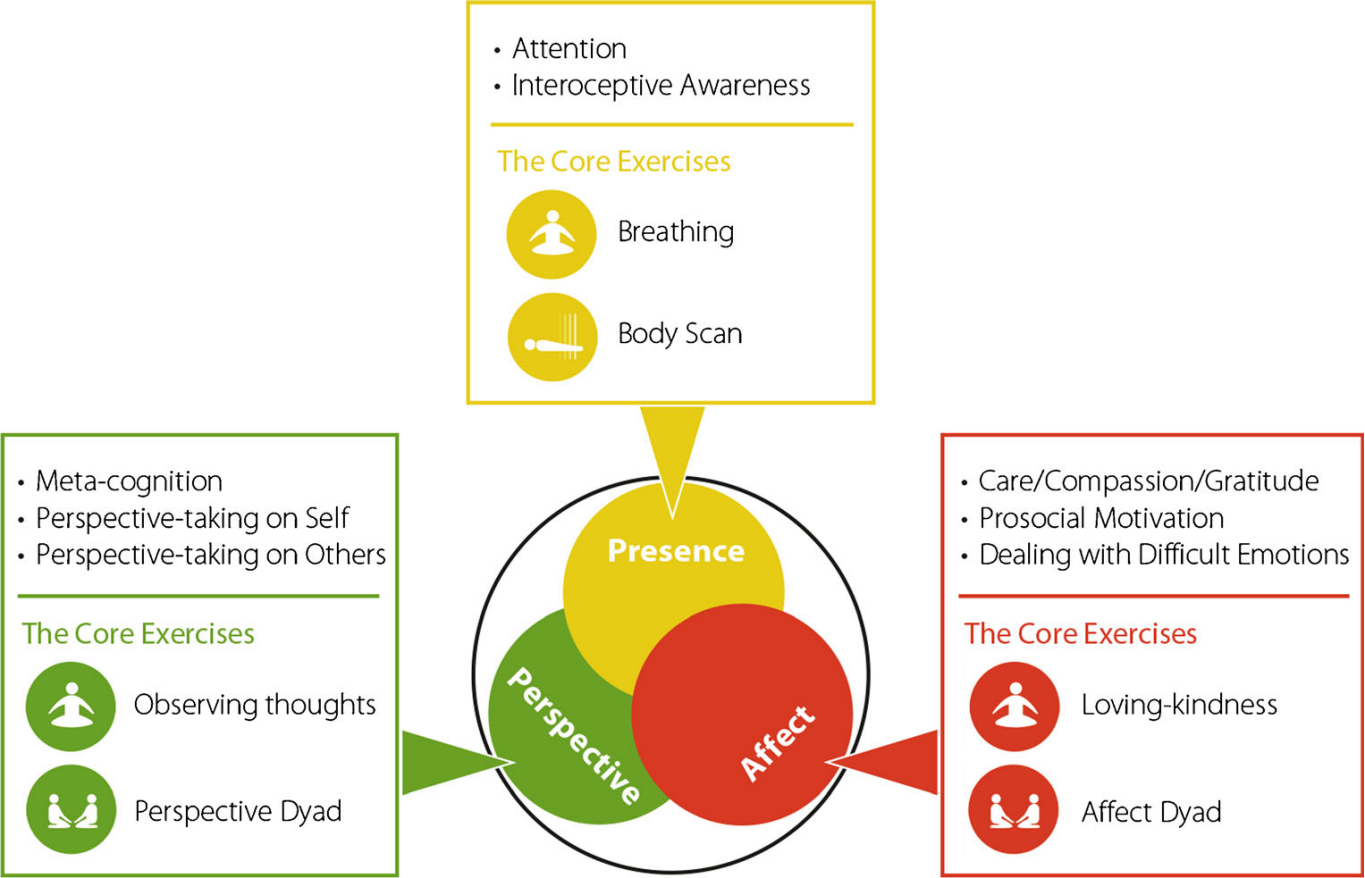
Overview of the aims and exercises per module in the ReSource Project. Reprinted from Singer et al. (2016)

**Fig. 2:**
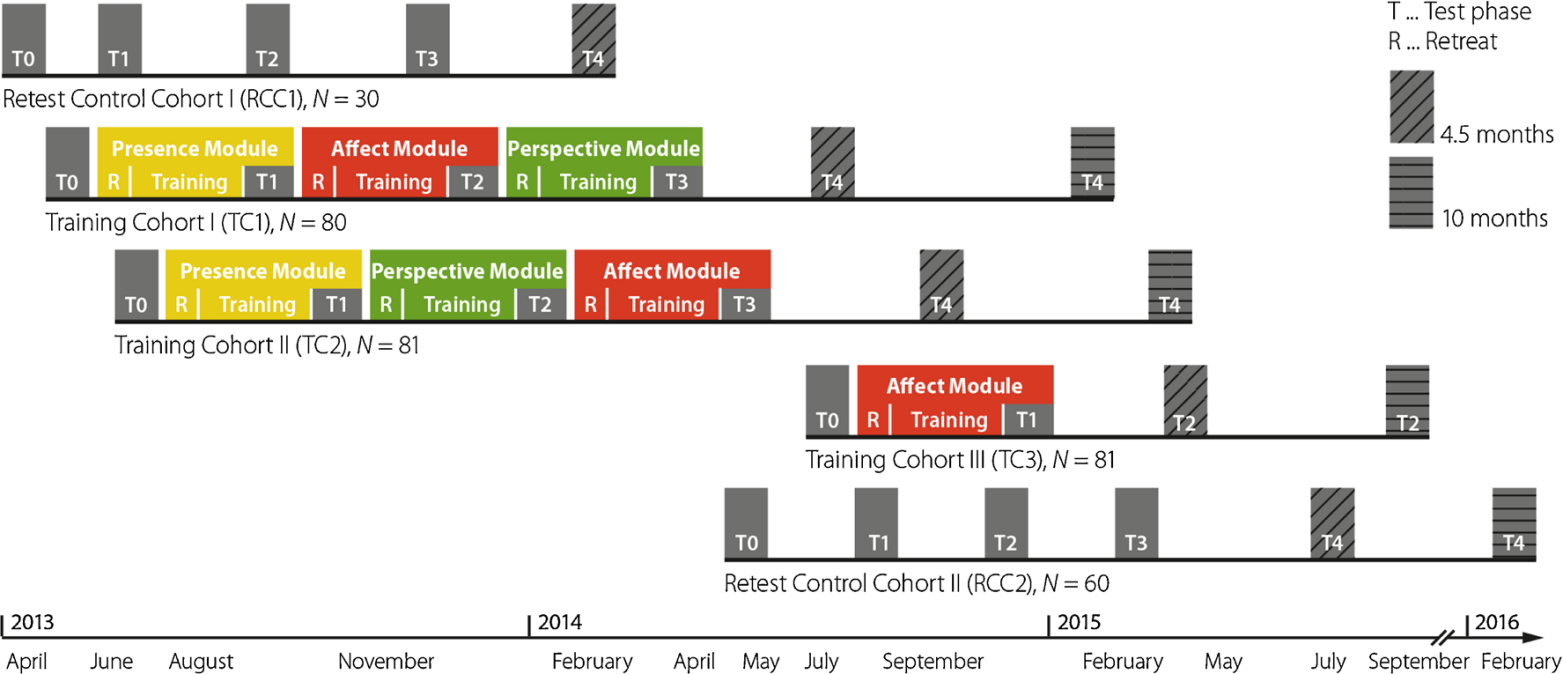
Timeline and study design of the ReSource Project. Follow-up measures (T4; or T2 for TC3) are not reported in this paper. Reprinted from Singer et al. (2016)

To shed light onto these questions, we tested the differential effects of different mental training practices on a number of facets of self-reported mindfulness and compassion scales. We did so in the context of the ReSource Project, a large-scale 9-month longitudinal training study (Singer et al. 2016), in which participants completed three distinct 3-month mental training modules (see Figs. 1 and 2). One training module, Presence, consisted of present-moment-focused attention and interoceptive awareness practices, with breathing meditation and the body scan as daily core practices. These practices are also at the core of mindfulness-based training programs such as MBSR (Kabat-Zinn 1982). Importantly, the ReSource Project teachers were explicitly told to not allude to explicit compassion-like states in their instructions for the Presence module. The Presence module was followed by two other training modules, the Affect and the Perspective modules. The Affect module trained socio-affective, motivational, and affiliative capacities, such as gratitude, compassion (including self-compassion), prosocial motivation, and watching and accepting difficult emotions, through loving-kindness meditation and a dyadic exercise as the two daily core practices. Thus, including two separate modules, the Presence and the Affect modules, enabled us to isolate and compare the effects of purely present-moment- and attention-focused mindfulness practices with those explicitly targeting affective qualities such as kindness and compassion. The Perspective module was aimed at cultivating socio-cognitive capacities such as meta-cognition and perspective-taking on self and others by incorporating core exercises like observing thoughts meditation and a daily dyadic perspective taking exercise on different aspects of the self and other people (for details, see Singer et al. 2016). The distinctions between these three modules were based not only on the different classifications of meditation practices taken in different Buddhist traditions (see, e.g., Gethin 1998; Lutz et al. 2007) or recently in contemplative sciences (Dahl et al. 2015; Lutz et al. 2008), but also on neuroscientific research which reveals a differentiation between different brain networks underlying (a) attentional processes (Petersen and Posner 2012), (b) socio-affective, and (c) socio-cognitive routes for understanding the self and others (de Vignemont and Singer 2006; Kanske et al. 2015; Singer 2012; for details about the rationale and theoretical backbone behind the ReSource Project, please see Singer et al. 2016, and see Fig. 1 for core practices of each module).

Based on the ReSource model (Singer et al. 2016), we expected differential effects of the three modules on different subscales of mindfulness, compassion, and self-compassion trait questionnaires. More specifically, we expected that (1) the Presence training module would be especially effective in increasing subscales related to attention and awareness to the present moment (i.e., presence, acting with awareness, observing) but would *not* necessarily lead to a cascade of improvements of ratings across nonjudgmental acceptance and compassion-based subscales. In line with the suggestion by Neff and Dahm (2015), we expected that compassion and self-compassion, but also ethical-motivational aspects of mindfulness, would benefit from specific, targeted cultivation of affective-motivational qualities and would not follow automatically from presence-focused training. Hence, we expected (2) the Affect module to specifically increase self-ratings of compassion, self-compassion, and also ratings on mindfulness subscales like acceptance and nonjudgment (see Fig. 1). In addition, we expected that (3) the Perspective module would most strongly increase ratings on subscales measuring observing and describing thoughts, as well as passive acceptance in the form of nonreactivity and equanimity. Finally, because the attentional capacities are often viewed as basic capacities that are prerequisites for but are also further cultivated in all other meditation-based practices (Rapgay and Bystrisky 2009; Wallace 2006), we also (4) expected improvements of the scales affected by the Presence module after the other two modules, Perspective and Affect.

## Methods

### Participants

The data presented here were collected as part of the ReSource Project, a large 9-month longitudinal study on the effects of mental training (for details, see Singer et al. 2016). Participants were thoroughly screened to exclude participants with health problems and previous meditation experience. In total, 332 participants (197 female, age range = 20–55, Mean_Age_ = 40.74, SD_Age_ = 9.24) took part in the study and were assigned to four different test cohorts (TC1 = 80, TC2 = 81, TC3 = 81, retest control cohort (RCC) = 90) that were matched on demographics and self-reported traits, including mindfulness (Five-Facet Mindfulness Questionnaire (FFMQ); Buchheld et al. 2001; Walach et al. 2006), self-compassion (Self-Compassion Scale (SCS); Neff 2003), and compassion (Compassion Scale (CS); Pommier 2011; for details, see Singer et al. 2016, p.48). An overview of the sample sizes available for every questionnaire reported here per time point, accounting for dropout and missing data, can be found in Table 1. Due to the multi-method approach of the ReSource Study, we did not conduct power analyses to determine the necessary sample size (as we would have had to designate one measure on which to base the analysis). Instead, we used large sample sizes per test cohort that exceed the typical sample size of these sort of interventions.

**Table 1:** Dropout and final samples per cohort, time point, and questionnaire

Questionnaire	RCC				TC1				TC2				TC3	
	T0	T1	T2	T3	T0	T1	T2	T3	T0	T1	T2	T3	T0	T1
Study														
Full sample	90				80				81				81	
Dropout^a^	5	6	8	11	2	3	4	8	0	5	5	6	1	5
FMI														
Missing^b^	0	0	3	0	3	3	0	0	0	1	0	0	0	0
Sample	85	84	79	79	75	74	76	72	81	75	76	75	80	76
FFMQ														
Missing^b^	0	0	4	0	3	3	0	0	0	1	0	0	0	0
Sample	85	84	78	79	75	74	76	72	81	75	76	75	80	76
SCS														
Missing^b^	0	0	4	0	3	3	0	0	0	1	0	0	0	0
Sample	85	84	78	79	75	74	76	72	81	75	76	75	80	76
CS														
Missing^b^	0	6	5	0	3	3	0	0	0	1	0	0	0	0
Sample	85	78	77	79	75	74	76	72	81	75	76	75	80	76
FoC														
Missing^b^	0	0	4	0	3	3	0	0	1	1	0	0	0	0
Sample	85	84	78	79	75	74	76	72	80	75	76	75	80	76

^a^ Cumulative dropout or exclusion due to medical reasons, discomfort with study or experiments, time constraints, or other (see Singer et al. 2016, for details)

^b^Missing data due to noncompliance

The study was registered at ClinicalTrials.gov (Protocol Registration System) under the title “Plasticity of the Compassionate Brain.” Ethical approval was given by the Research Ethics Committees of the University of Leipzig (376/12-ff) and the Humboldt University in Berlin (2013-02, 2013-29, 2014-10). All participants gave written informed consent.

### Procedure

The ReSource Project was a modular longitudinal study (see also Fig. 2) that consisted of four test cohorts, which completed three different training modules.

#### Modules

The Presence module was designed to cultivate present-moment awareness and attention, and interoceptive awareness. The daily core exercises were breathing meditation and body scan, and during the weekly sessions, participants were introduced to other present-moment-focused and attention-based meditations such as focusing on taste, sound, or visual objects, which made this module most similar to mindfulness-based programs such the MBSR (Kabat-Zinn 1982). Because such present-moment and attention-focused practices are often seen as the basis for other contemplative practices (Rapgay and Bystrisky 2009; Wallace 2006), the Presence module was implemented as the first module in the two main training cohorts (TC1 and TC2, see below).

The Affect module was aimed at cultivating socio-affective and motivational skills such as loving-kindness, gratitude and compassion, prosocial motivation, and the ability to observe and accept difficult emotions. Loving-kindness meditation (Salzberg 1995) and a newly developed contemplative affect dyads were implemented as daily core exercises (for further information, see Singer et al. 2016). During the affect dyad, the participants were paired with a partner and spent 5 min describing a situation they found difficult or were grateful for (or listening to their partner’s description) and then reversing roles. This exercise was included to specifically boost socio-affective skills, like empathy, compassion, and dealing with difficult emotions.

The Perspective module was aimed at cultivating meta-cognitive skills such as becoming aware of the content and the nature of one’s thoughts and becoming aware of different aspects of one’s own personality. It also focused on perspective-taking on others’ minds (i.e., Theory of Mind or mentalizing ability; Premack and Woodruff 1978). To train this socio-cognitive route, participants practiced two core exercises, “observing thoughts meditation” and a newly developed perspective dyad, on a daily basis. The perspective dyad consisted of describing (or listening to the partner’s description of) a situation from the perspective of one of one’s own inner parts. The respective practices are explained in more detail in Singer et al. (2016), but see Fig. 1 for an overview on the two core exercises per module.

#### Training Cohorts

The two main training cohorts, TC1 and TC2, participated in three different 13-week training modules (Presence, Affect, and Perspective). The order of the training modules differed between these two cohorts: TC1 trained in the order “Presence-Affect-Perspective,” while TC2 underwent the order “Presence-Perspective-Affect.” Thus, at T2 and T3, the two cohorts served as active control groups to each other. The third training cohort, TC3, only completed the Affect module serving as a control group for the presence modules performed by TC1 and TC2. Finally, a RCC (divided into two testing sequences for practical scheduling reasons) did not follow any training but was only tested in all measures. This design allowed us to compare the specific effect of each different module with each other, as well as with the retest control group.

The training modules for the three test cohorts generally began with a 3-day intensive retreat and continued with 13 weeks of individual daily practice at home accompanied by a weekly 2-h group session with teachers. The first 8 weeks of every module were designated to develop the practice, whereas the last 5 weeks consisted of repetition and deepening of the practices learned. These last 5 weeks were also the testing periods, which means that TC1 and TC2 were tested three times (T1–T3) in addition to the baseline testing before any training (T0), and TC3 was tested twice (T0 and T1). The RCC was also tested four times with a distance of 2–3 months between testing sessions to match the timeline of the training cohorts. In addition, all participants could voluntarily participate in a follow-up testing session (T4; these data are not reported here but will be reported elsewhere as part of a separate analysis on the long-term effects of mental training). During the full 5-week testing period at the end of each training module, the questionnaires included here were available to the participants on an online platform and could be filled out at convenient times from their homes. For an overview of the overall design and measurement periods of the study, see Fig. 2.

### Measures

For the purpose of the present paper, we included all questionnaires related to mindfulness, compassion, and self-compassion that we assessed in the ReSource Project. Mindfulness was assessed with the FMI and the Five-Facet Mindfulness Questionnaire (FFMQ). Compassion and self-compassion were measured using the SCS, the CS, and the Fear of Compassion Scale (FoC). Cronbach’s alphas as a measure of internal consistency of all subscales can be found in Table 2. Another questionnaire that is tangentially related to the topic of this paper and that we also assessed in the ReSource Project is the Interpersonal Reactivity Index (IRI; Davis 1983), which is a measure of empathy. Because empathy is not the focus of this paper, we included the results of the IRI in the supplementary materials (Table S2 and Fig. S1) for the interested reader.

**Table 2:** Cronbach’s alphas per time point and subscale

	Original	T0	T1	T2	T3
FMI					
Presence	0.78^a^	0.79	0.75	0.80	0.78
Acceptance	0.81^a^	0.71	0.69	0.73	0.70
FFMQ					
Observing	0.83^b^	0.82	0.82	0.83	0.85
Describing	0.91^b^	0.91	0.92	0.90	0.92
Nonreacting	0.75^b^	0.87	0.84	0.85	0.87
Acting with awareness	0.87^b^	0.85	0.83	0.85	0.89
Nonjudging	0.87^b^	0.89	0.90	0.90	0.92
SCS					
Self-kindness	0.78^c^	0.83	0.86	0.88	0.90
Self-judgment	0.77^c^	0.81	0.83	0.84	0.84
Common humanity	0.80^c^	0.65	0.75	0.76	0.76
Isolation	0.79^c^	0.80	0.80	0.84	0.83
Mindfulness	0.75^c^	0.70	0.73	0.74	0.76
Overidentification	0.81^c^	0.70	0.70	0.73	0.72
CS					
Kindness	0.77^d^	0.71	0.74	0.79	0.74
Indifference	0.68^d^	0.65	0.74	0.68	0.73
Common	0.70^d^	0.60	0.73	0.61	0.70
Separation	0.64^d^	0.64	0.72	0.68	0.67
Mindfulness	0.67^d^	0.59	0.65	0.63	0.63
Disengagement	0.57^d^	0.58	0.63	0.61	0.56
FoC					
Expressing	0.84/0.78^e^	0.85	0.87	0.90	0.88
Responding	0.85/0.87^e^	0.89	0.88	0.87	0.90
Self	0.92/0.85^e^	0.88	0.89	0.90	0.90

^a^Rasch reliability as reported in Sauer et al. (2011)

^b^Cronbach's α as reported in Baer et al. (2006)

^c^Cronbach's α as reported in Neff (2003)

^d^Cronbach's α as reported in Pommier (2011)

^e^Cronbach's α for students/therapists as reported in Gilbert et al. (2011)

#### Freiburg Mindfulness Inventory

The FMI was developed based on a definition of mindfulness of experienced meditators (Buchheld et al. 2001; Walach et al. 2006). The FMI was subsequently also tested in nonmeditators and reduced to a short version (Kohls et al. 2009), which is used here. The short version has been shown to be represented by two dimensions, *presence* and *acceptance* (Sauer et al. 2011). We used this questionnaire here because of its strong theoretical basis and because it matches the two broad categories of mindfulness proposed by Bishop et al. (2004).

#### Five-Facet Mindfulness Questionnaire

The FFMQ (Baer et al. 2006) was constructed to address the lack in consensus of an operationalization of mindfulness by combining a number of existing mindfulness scales in a factor analysis to extract meaningful dimensions of mindfulness. This resulted in five factors: *nonreacting* to inner experiences, *observing* inner experiences, *acting with awareness*, *describing*, and *nonjudging* of experience. We used this questionnaire here because it is grounded in the existing operationalizations and because it provides fine-grained facets of mindfulness.

#### Self-Compassion Scale

The SCS (Neff 2003; Raes et al. 2011) consists of six subscales: *self-kindness*, *self-judgment*, *common humanity*, *isolation*, *mindfulnes*s, and *overidentification*. Note that these subscales represent pairs of opposing constructs, e.g., *overidentification* is supposed to measure the opposite of *mindfulness*, and *self-judgment* and *isolation* are measures of the absence of *self-kindness* and *common humanity*, respectively.

#### Compassion Scale

The CS (Neff 2003; Raes et al. 2011; Pommier 2011) has similar subscales as the SCS but is directed at how respondents relate to others’ suffering. The CS also consists of six subscales: *kindness*, *indifference*, *common*, *separation*, *mindfulness*, and *disengagement*. Similar to the SCS, these subscales represent also pairs of opposing constructs.

#### Fear of Compassion Scale

The FoC (Gilbert et al. 2011) measures compassion by assessing the ratings on negatively phrased items, i.e., the absence of compassion. The three subscales are fear of expressing compassion for others (*expressing*), fear of responding to compassion from others (*responding*), and fear of experiencing self-compassion (*self*).

### Data Analyses

The responses on the questionnaires were recorded and summed (FFMQ) or averaged (FMI, SCS, CS, FoC) according to the questionnaires’ protocols, which resulted in one score per participant and time point for each of the 22 subscales. As illustrated in Fig. 2, we analyzed four time points (T0–T3) for all groups except TC3 who were only tested at two time points. To test whether the different modules led to differential changes in these different facets of mindfulness and (self-)compassion, we used these scores as dependent variables in separate linear mixed models per subscale (Baayen 2008), which we fitted using the lme() function of the nlme package (Pinheiro et al. 2016) in R (R Core Team 2016). As fixed effect predictor, we used a factor that combined time point and cohort, e.g. “T0_TC1” as a factor level representing test cohort 1 (TC1) at time point 0 (baseline, T0). We chose this model so that we could include, in addition to a random intercept for participants, a first-order autoregressive correlation structure (AR1) to account for time in our longitudinal design. This is only possible with the lme() function in R, but because this function does not work with an unbalanced design, we used an interaction factor. However, we also conducted more traditional analyses (omitting the AR1), and the general pattern of results is the same. These results can be found in the supplementary materials (Table S1). In addition, we included gender and age (normalized with a z transform) as covariates.

For every subscale, we first conducted a full-null model comparison to test the overall effect of the interaction of time point and module. The null model consisted of the same random effect and correlation structure, as well as the control variables gender and age, but excluded the fixed effect (Forstmeier and Schielzeth 2011).

Full model:

$$ {\mathrm{Score}}_{\mathrm{subscale}}={\beta}_0+{\beta_1}^{\ast}\mathrm{gender}+{\beta_2}^{\ast}\mathrm{age}+{\beta_3}^{\ast}\left(\mathrm{module}\ \mathrm{by}\ \mathrm{time}\ \mathrm{point}\right)+{\mathrm{random}\ \mathrm{effect}}_{\mathrm{participant}} $$

Null model:

$$ {\mathrm{Score}}_{\mathrm{subscale}}={\beta}_0+{\beta_1}^{\ast}\mathrm{gender}+{\beta_2}^{\ast}\mathrm{age}+{\mathrm{random}\ \mathrm{effect}}_{\mathrm{participant}} $$

Comparing these two models allowed us to assess whether including the different modules by time point combinations explained variance in the dependent scores beyond participant-specific differences. The comparison was carried out using a likelihood ratio test (Dobson and Barnett 2008). We used the *p* values obtained in these separate tests to correct for multiple testing (using Holm’s (1979) stepwise family-wise error correction method and the p.adjust function in R) across all subscales. These corrected *p* values are reported throughout the text as *p*_cor_. We also obtained marginal and conditional *R* ^2^-like effect sizes for the full models by dividing the variance of the fixed effects (excluding gender and age in this model; and including the random effects for the conditional *R* ^2^s) by the sum of the variances of the fixed and random effects and the residuals (Nakagawa and Schielzeth 2013). For linear mixed models, the marginal effect sizes of the fixed effects are generally rather small, as usually a great amount of the variance in the dependent variable is explained by individual differences, i.e., the random effects. In addition, we calculated effect sizes for the change per module and time point as compared to the RCC according to the suggestion of Morris (d_ppc2_, 2008) for pretest-posttest-control group designs. Since these effect sizes are not specifically suggested for linear mixed models, we only report in the “Results” section those that are relatively big. All of these effect sizes, however, can be found in Table 10.

If the full-null model comparison reached significance, we concluded that the module by time point variable added explanatory value and extracted planned contrasts (using the glht() function of the multcomp package, Hothorn et al. 2008) between the different modules per time point from the model. In particular, because we wanted to compare the groups at the matching time points, we used custom contrasts of the interaction, e.g., we contrasted the scores of the participants after the Presence module, i.e., at T1, with the change scores of the RCC also only at T1. In addition, we also calculated contrasts of the average module effect, i.e., we collapsed over the two (when testing Perspective) or three (when testing Affect) time points when coding the contrasts for the overall effects of the different modules. The reason for this approach is to account for the matching retest effects and to avoid deflating or inflating the effects by averaging over nonmatching time points. Importantly, because we were interested in the change between two time points, we extracted change contrasts (e.g., (T2_TC1-T1_TC1) – (T2_RCC – T1_RCC) comparing the change in cohort TC1 between time points T1 and T2 with the same change in the control group).

As a visual summary, we included pie charts scaled to the *R* ^2^-like effect sizes, in combination with example items for each subscale. The sizes of the slices of the pie charts represent the absolute average estimate of the specific module compared to the RCC. These plots can be found in Figs. 6, 7, 8, and 9.

## Results

### Presence (Subscale of the FMI)

**Fig. 3:**
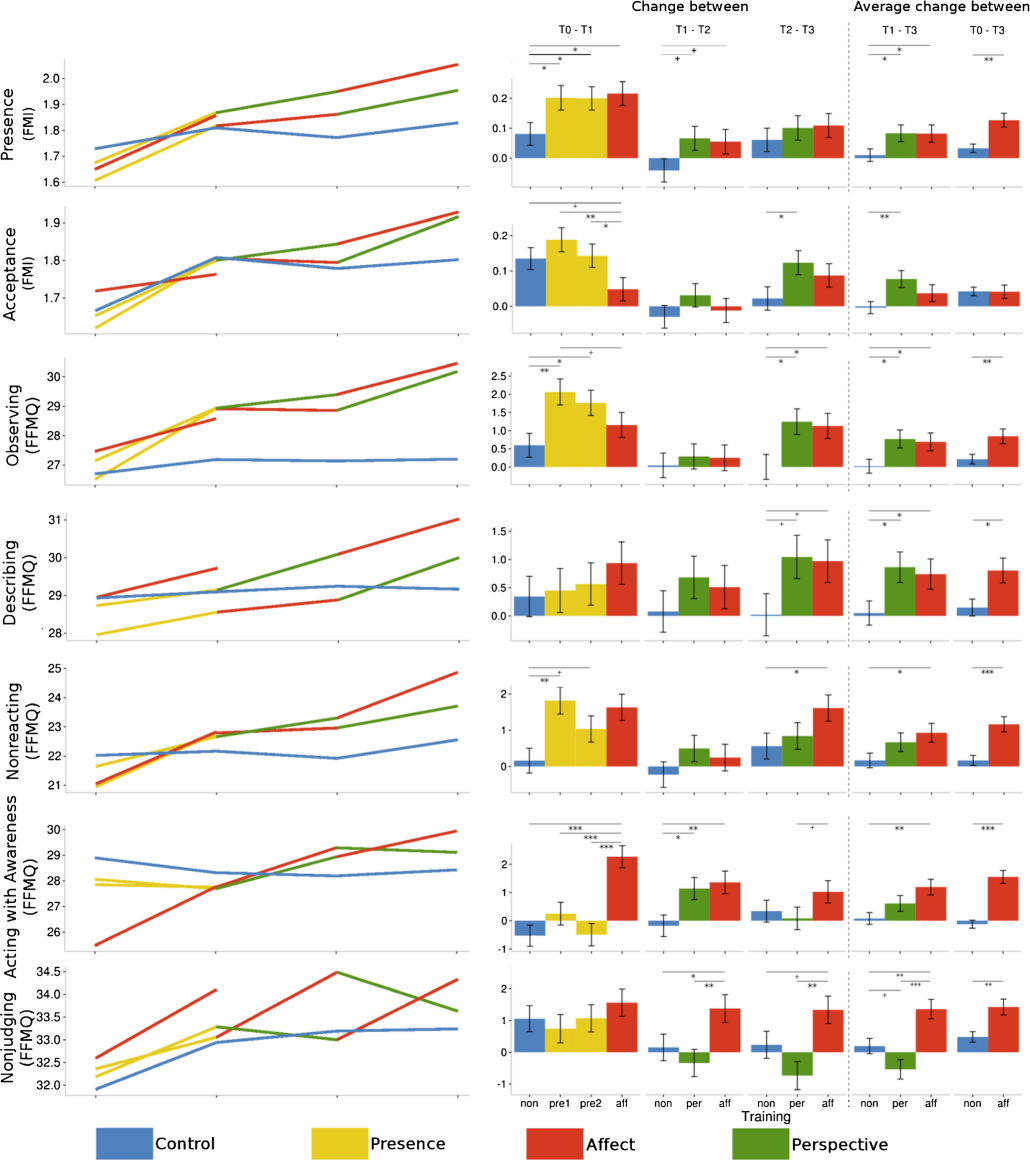
Averaged raw data per cohort and time point (*left*) and estimates and standard errors of the changes between time points derived from the contrasts of the linear mixed models (*right*) per subscale of the *Freiburg Mindfulness Inventory* and the *Five-Facet Mindfulness Questionnaire*. The three *leftmost bar charts* represent the change between two subsequent time points, whereas the two *right bar chart columns* represent the average change for both Perspective modules (plus the matching control and Affect modules) and the average change for all three Affect modules (plus the matching control cohorts). +*p* < .1; *p ≤ .05; **p ≤ .01; ***p ≤ .001

The full model predicted the variance in the scores significantly better than the null model (likelihood ratio test: *χ* ^*2*^ = 159.03, *df* = 13, *p* < 0.001, *p*_cor_ < 0.001, *R* ^2^_marg_ = 0.063, *R* ^2^_cond_ = 0.677; see Fig. 3 for scores and Fig. 6 for a visual summary). Thus, the self-reported scores in the subscale called *presence* (FMI) differed between the different module by time point combinations. Planned contrasts (see Tables 3 and 10 for effect sizes) revealed that after the Presence modules, changes in the ratings of *presence* on the FMI were greater than in the retest control group, but not significantly different from the changes after the Affect module at T1. At T2, both the Affect and Perspective modules led to marginally greater increases in ratings than the retest control group, whereby these two training modules did not differ from each other. At T3, neither the changes after the Affect nor after the Perspective module were significantly different from the retest control cohort and the changes in the two training cohorts also did not differ from each other. When averaging over the changes to T2 and T3, both the Affect and the Perspective trainings led to greater changes than the RCC, while the effects of the two training modules did not differ. Over all three time points, the changes in ratings after the Affect modules differed significantly from the retest effect in the RCC. Thus, the Presence, as well as the Affect module at T1, clearly led to increases in *presence* ratings, while the Perspective and Affect modules at later time points continued to lead to slight increases.

**Table 3:** Results of pairwise comparison contrasts derived from the linear mixed model of the effects of the different trainings and time points on the change scores of the subscales of the Freiburg Mindfulness Inventory

Contrast	Presence			Acceptance		
	*β* ± SE	*Z*	*p*	*β* ± SE	*Z*	*p*
Change between T0 and T1						
Affect-RCC	0.14 ± 0.03	2.47	.014	−0.09 ± 0.05	−1.91	.056
Pres (both)-RCC	0.12 ± 0.05	2.55	.011	0.03 ± 0.04	0.79	.430
Pres (TC1)-RCC	0.12 ± 0.06	2.18	.029	0.05 ± 0.05	1.16	.248
Pres (TC2)-RCC	0.12 ± 0.06	2.19	.029	0.01 ± 0.05	0.18	.854
Pres (both)-Affect	−0.02 ± 0.05	−0.30	.763	0.12 ± 0.04	2.91	.004
Pres (TC1)-Affect	−0.01 ± 0.06	−0.24	.812	0.14 ± 0.05	2.96	.003
Pres (TC2)-Affect	−0.02 ± 0.06	−0.28	.777	0.10 ± 0.05	2.06	.040
Change between T1 and T2						
Affect-RCC	0.10 ± 0.06	1.72	.085	0.02 ± 0.05	0.38	.708
Persp-RCC	0.11 ± 0.06	1.92	.055	0.06 ± 0.05	1.33	.185
Persp-Affect	0.01 ± 0.06	0.18	.859	0.05 ± 0.05	0.92	.355
Change between T2 and T3						
Affect-RCC	0.05 ± 0.06	0.86	.389	0.06 ± 0.05	1.38	.166
Persp-RCC	0.04 ± 0.06	0.70	.482	0.10 ± 0.05	2.16	.031
Persp-Affect	−0.01 ± 0.06	−0.15	.881	0.04 ± 0.05	0.78	.437
Change between T1 and T3						
Affect-RCC	0.07 ± 0.04	2.05	.040	0.04 ± 0.03	1.40	.163
Persp-RCC	0.07 ± 0.04	2.08	.038	0.08 ± 0.03	2.77	.006
Persp-Affect	0.00 ± 0.05	0.02	.987	0.04 ± 0.04	1.02	.310
Change between T0 and T3						
Affect-RCC	0.09 ± 0.03	3.45	.001	−0.00 ± 0.02	−0.08	.940

*Pres* Presence module, *Persp* Perspective module, *Affect* Affect module, *RCC* retest control cohort

### Acceptance (FMI)

The full model was significantly better than the null model (*χ* ^*2*^ = 132.14, *df* = 13, *p* < 0.001, *p*_cor_ < 0.001, *R* ^2^_marg_ = 0.043, *R* ^2^_cond_ = 0.687, see Fig. 3 for scores and Fig. 6 for a visual summary). The change in ratings on the *acceptance* subscale after the Presence modules did not differ significantly from the retest control group’s change. However, the Affect module (at T1) led to smaller increases in ratings compared to the Presence module and—marginally—compared to the retest control cohort. Averaging over the Perspective modules showed an increase in *acceptance* ratings compared to the control group but not when compared to the Affect modules. This effect seems to be driven by the change between T2 and T3. Ratings after the Affect modules averaged over all time points did not significantly differ from those in the control group (see Table 3 for the pairwise contrasts comparing the modules). Therefore, *acceptance* ratings were increased after the Perspective module only.

### Observing (FFMQ)

The full-null model comparison was significant (*χ* ^2^ = 141.27, *df* = 13, *p* < 0.001, *p*_cor_ < 0.001, *R* ^2^_marg_ = 0.053, *R* ^2^_cond_ = 0.755, see Fig. 3 for scores and Fig. 6 for a visual summary). The Presence modules caused increases in *observing* beyond changes found in the control cohort. Average changes after the Perspective modules, as well as the Affect modules, were greater than those found in the control group, but the two module groups did not differ from each other. Looking separately at T1–T2 and T2–T3, this effect was found only in the change between T2 and T3. Changes in ratings after all three Affect modules averaged increased more than those in the control group (see Table 4 for the results of the contrasts). The ratings on the *observing* subscale were thus increased by all three modules, but not at all time points.

**Table 4:** Results of pairwise comparison contrasts derived from the linear mixed model of the effects of the different trainings and time points on the change scores of the subscales of Five-Facet Mindfulness Questionnaire

Contrast	Observing			Describing			Nonreacting			Acting with awareness			Nonjudging		
	*β* ± SE	*Z*	*p*	*β* ± SE	*Z*	*p*	*β* ± SE	*Z*	*p*	*β* ± SE	*Z*	*p*	*β* ± SE	*Z*	*p*
Change between T0 and T1															
Affect-RCC	0.60 ± 0.48	1.16	.245	0.60 ± 0.52	1.14	.255	1.47 ± 0.50	2.95	.003	2.80 ± 0.54	5.19	.000	0.51 ± 0.59	0.85	.394
Pres (both)-RCC	1.32 ± 0.41	3.20	.001	0.16 ± 0.45	0.37	.715	1.27 ± 0.43	2.94	.003	0.41 ± 0.47	0.88	.380	−0.15 ± 0.51	−0.29	.771
Pres (TC1)-RCC	1.47 ± 0.49	3.06	.003	0.11 ± 0.53	0.20	.843	1.66 ± 0.51	3.26	.001	0.78 ± 0.55	1.42	.156	−0.31 ± 0.60	−0.52	.605
Pres (TC2)-RCC	1.17 ± 0.48	2.45	.014	0.22 ± 0.52	0.43	.668	0.88 ± 0.50	1.76	.079	0.04 ± 0.54	0.07	.945	0.01 ± 0.59	0.02	.981
Pres (both)-Affect	0.76 ± 0.43	1.78	.074	−0.43 ± 0.46	−0.92	.357	−0.20 ± 0.45	−0.46	.648	−2.39 ± 0.48	−4.96	.000	−0.65 ± 0.53	−1.23	.217
Pres (TC1)-Affect	0.91 ± 0.50	1.83	.068	−0.49 ± 0.54	−0.90	.369	0.19 ± 0.52	0.36	.719	−2.02 ± 0.56	−3.59	.000	−0.82 ± 0.62	−1.32	.187
Pres (TC2)-Affect	0.61 ± 0.50	1.25	.211	−0.37 ± 0.53	−0.69	.488	−0.59 ± 0.51	−1.16	.245	−2.76 ± 0.55	−5.00	.000	−0.49 ± 0.61	−0.81	.420
Change between T1 and T2															
Affect-RCC	0.21 ± 0.50	0.43	.665	0.43 ± 0.53	0.81	.416	0.47 ± 0.51	0.93	.354	1.54 ± 0.55	2.80	.005	1.23 ± 0.61	2.02	.043
Persp-RCC	0.25 ± 0.48	0.51	.608	0.60 ± 0.53	1.15	.251	0.72 ± 0.51	1.43	.152	1.32 ± 0.55	2.42	.016	−0.49 ± 0.60	−0.82	.411
Persp-Affect	0.04 ± 0.50	0.07	.941	0.17 ± 0.54	0.32	.749	0.25 ± 0.52	0.49	.627	−0.22 ± 0.56	−0.40	.692	−1.72 ± 0.61	−2.80	.005
Change between T2 and T3															
Affect-RCC	1.13 ± 0.49	2.31	.021	0.95 ± 0.53	1.79	.073	1.05 ± 0.51	2.06	.039	0.69 ± 0.55	1.25	.212	1.10 ± 0.61	1.82	.069
Persp-RCC	1.25 ± 0.49	2.53	.011	1.02 ± 0.54	1.91	.056	0.28 ± 0.51	0.54	.588	−0.25 ± 0.56	−0.46	.648	−0.97 ± 0.61	−1.59	.113
Persp-Affect	0.12 ± 0.50	0.24	.813	0.07 ± 0.54	0.14	.890	−0.77 ± 0.52	−1.49	.135	−0.94 ± 0.56	−1.68	.092	−2.07 ± 0.62	−3.37	.001
Change between T1 and T3															
Affect-RCC	0.67 ± 0.31	2.14	.032	0.69 ± 0.34	2.01	.045	0.76 ± 0.33	2.33	.020	1.12 ± 0.35	3.19	.001	1.16 ± 0.39	2.98	.003
Persp-RCC	0.75 ± 0.31	2.39	.017	0.81 ± 0.34	2.37	.018	0.50 ± 0.33	1.53	.126	0.53 ± 0.35	1.53	.127	−0.73 ± 0.39	−1.88	.061
Persp–Affect	0.08 ± 0.41	0.19	.849	0.12 ± 0.43	0.28	.776	−0.26 ± 0.42	−0.62	.537	−0.58 ± 0.46	−1.26	.210	−1.90 ± 0.50	−3.79	.000
Change between T0 and T3															
Affect-RCC	0.63 ± 0.24	2.62	.009	0.67 ± 0.27	2.48	.013	1.00 ± 0.25	3.96	.000	1.68 ± 0.27	6.24	.000	0.94 ± 0.30	3.14	.002

*Pres* Presence module, *Persp* Perspective module, *Affect* Affect module, *RCC* retest control cohort

### Describing (FFMQ)

Including the combination of modules and time points in the full model predicted the scores on the *describing* subscale better than only accounting for participant-specific variables and random intercepts (*χ* ^2^ = 45.58, *df* = 13, *p* < 0.001, *p*_cor_ < 0.001, *R* ^2^_marg_ = 0.016, *R* ^2^_cond_ = 0.721; see Fig. 3 for scores, Fig. 6 for a visual summary and Table 4 for the estimates of the contrasts). At the different time points, the changes between the training cohorts did not differ significantly from the RCC’s changes in scores. Averaging over the two Perspective modules, as well as over the matching or all Affect modules revealed that those significantly differed from the RCC, but not from each other. Thus, both the Perspective and the Affect module seem to lead to slight increases in self-ratings on the *describing* subscale compared to the RCC.

### Nonreacting (FFMQ)

The full-null model comparison was only marginally significant after correction for multiple testing (*χ* ^2^ = 116.82, *df* = 13, *p* < 0.001, *p*_cor_ < 0.001, *R* ^2^_marg_ = 0.050, *R* ^2^_cond_ = 0.622; see Fig. 3 for scores and Fig. 6 for a visual summary). Ratings on the *nonreacting* subscale increased significantly more after the Presence (although only a trend in TC2) and the Affect modules than in the control group, but the two groups did not differ. The Perspective module did not result in significantly different changes in scores than the control group or the Affect groups. Averaging over all Affect modules revealed a greater increase through the Affect module than in the RCC (see Table 4 for the results of the contrasts). *Nonreacting* scores were hence increased following the Affect and the Presence modules.

### Acting with Awareness (FFMQ)

Including time point and module had a significant effect on the change scores of *acting with awareness* (*χ* ^2^ = 90.13, *df* = 13, *p* < 0.001, *p*_cor_ < 0.001, *R* ^2^_marg_ = 0.039, *R* ^2^_cond_ = 0.700; see Fig. 3 for scores and Fig. 6 for a visual summary). Pairwise comparisons (see Table 4) showed that Affect, but not Presence, led to higher increases in ratings on the *acting with awareness* subscale than the retest control cohort at T1, which is a medium effect (see Table 9). The ratings increased significantly more after both the Perspective and Affect modules between T1 and T2, and the increases between those two module cohorts did not differ significantly. In contrast, changes between T2 and T3 after both the Presence and Affect modules were not significantly different from the retest control cohort’s changes. However, the changes in those two module cohorts differed slightly from each other because ratings in the Affect cohort further increased while those in the Perspective cohort decreased. When averaged over time points, also only the Affect module significantly differed from the RCC. Thus, especially the Affect module led to increases in ratings on the *acting with awareness* subscale.

### Nonjudging (FFMQ)

The full model explained the changes in *nonjudging* better than the null model (*χ* ^2^ = 60.98, *df* = 13, *p* < 0.001, *p*_cor_ < 0.001, *R* ^2^_marg_ = 0.020, *R* ^2^_cond_ = 0.642; see Figs. 3 and 6). Contrasts (see Table 4) revealed that at T1, neither the Presence nor Affect modules led to significantly greater change in ratings that the RCC. At both T2 and T3, Affect in general increased ratings as compared to Perspective and the control group (marginally at T3) at the matching time points, whereas changes after the Perspective modules did not differ from the RCC’s changes (although there is a trend when averaged over time points). Thus, the training in the Affect module clearly led to increases in *nonjudging* ratings, especially when compared to the rather decreasing effect of the Perspective module.

### Self-Kindness (SCS)

**Fig. 4:**
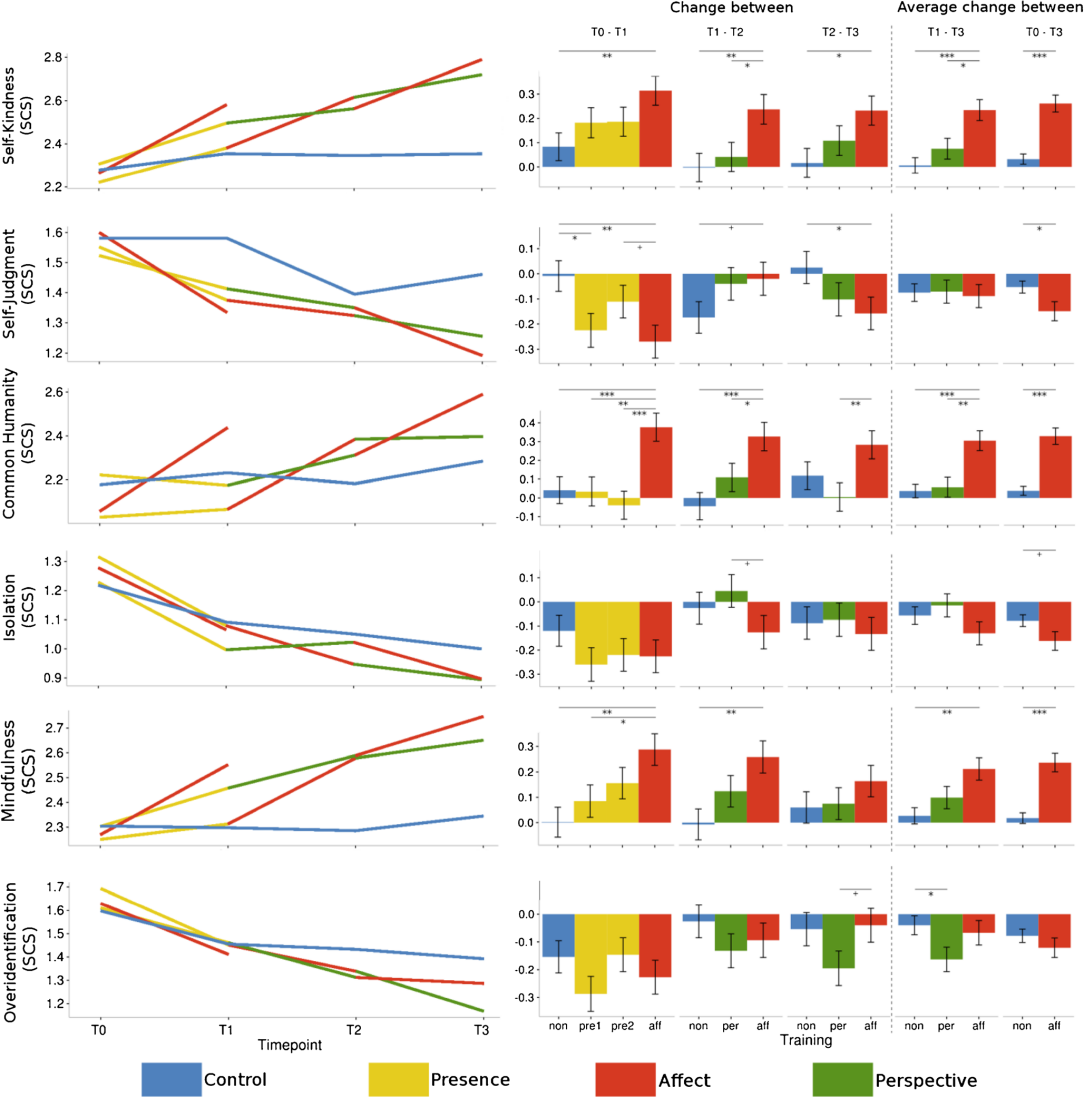
Averaged raw data per cohort and time point (*left*) and estimates and standard errors of the changes between time points derived from the contrasts of the linear mixed models (*right*) per subscale of the *Self-Compassion Scale*. The three *leftmost bar charts* represent the change between two subsequent time points, whereas the two *right bar chart columns* represent the average change for both Perspective modules (plus the matching control and Affect modules) and the average change for all three Affect modules (plus the matching control cohorts). +*p* < .1; *p ≤ .05; **p ≤ .01; ***p ≤ .001

Including the combination of module and time point improved the model fit significantly (*χ* ^2^ = 129.88, *df* = 13, *p* < 0.001, *p*_cor_ < 0.001, *R* ^2^_marg_ = 0.056, *R* ^2^_cond_ = 0.675). Changes after the Presence modules did not differ significantly from the RCC or the Affect module. Similarly, the Perspective module did not differ from the control group, but resulted in smaller increases than the Affect module especially at T2. In general, the changes after the Affect modules were greater than the change in the control group participants (see Figs. 4 and 7, and Table 5 for contrasts). Self-kindness was thus clearly increased after the Affect modules compared to the Perspective modules and the RCC.

**Table 5:** Results of pairwise comparison contrasts derived from the linear mixed model of the effects of the different trainings and time points on the change scores of the subscales of the Self-Compassion Scale

Contrast	Self-kindness			Self-judgment			Common humanity		
	*β* ± SE	*Z*	*p*	*β* ± SE	*Z*	*p*	*β* ± SE	*Z*	*p*
Change between T0 and T1									
Affect-RCC	0.23 ± 0.08	2.78	.005	−0.26 ± 0.09	−2.92	.004	0.34 ± 0.10	3.26	.001
Pres (both)-RCC	0.10 ± 0.07	1.41	.159	−0.16 ± 0.08	−2.06	.039	−0.04 ± 0.09	−0.49	.623
Pres (TC1)-RCC	0.10 ± 0.09	1.17	.242	−0.22 ± 0.09	−2.37	.018	−0.01 ± 0.11	−0.07	.943
Pres (TC2)-RCC	0.10 ± 0.08	1.24	.214	−0.10 ± 0.09	−1.15	.252	−0.08 ± 0.10	−0.78	.437
Pres (both)-Affect	−0.13 ± 0.07	−1.76	.079	0.10 ± 0.08	1.28	.202	−0.38 ± 0.09	−4.13	.000
Pres (TC1)-Affect	−0.13 ± 0.09	−1.53	.127	0.05 ± 0.09	0.48	.629	−0.34 ± 0.11	−3.20	.001
Pres (TC2)-Affect	−0.13 ± 0.09	−1.51	.132	0.16 ± 0.09	1.73	.083	−0.42 ± 0.11	−3.94	.000
Change between T1 and T2									
Affect-RCC	0.24 ± 0.09	2.82	.005	0.15 ± 0.09	1.69	.091	0.37 ± 0.11	3.53	.000
Persp-RCC	0.04 ± 0.08	0.52	.601	0.13 ± 0.09	1.48	.139	0.15 ± 0.11	1.46	.143
Persp-Affect	−0.20 ± 0.09	−2.27	.023	−0.02 ± 0.09	−0.22	.826	−0.22 ± 0.11	−2.04	.041
Change between T2 and T3									
Affect-RCC	0.22 ± 0.09	2.55	.011	−0.18 ± 0.09	−1.99	.046	0.17 ± 0.11	1.56	.118
Persp-RCC	0.09 ± 0.09	1.08	.279	−0.13 ± 0.09	−1.37	.171	−0.11 ± 0.11	−1.07	.287
Persp-Affect	−0.12 ± 0.09	−1.43	.152	0.06 ± 0.09	0.60	.546	−0.28 ± 0.11	−2.60	.009
Change between T1 and T3									
Affect-RCC	0.23 ± 0.05	4.26	.000	−0.01 ± 0.06	−0.24	.810	0.27 ± 0.07	4.14	.000
Persp-RCC	0.09 ± 0.09	1.28	.202	0.00 ± 0.06	0.07	.946	0.02 ± 0.07	0.31	.757
Persp-Affect	−0.12 ± 0.09	−2.22	.027	0.02 ± 0.08	0.23	.817	−0.25 ± 0.09	−2.69	.007
Change between T0 and T3									
Affect-RCC	0.23 ± 0.05	5.57	.000	−0.10 ± 0.04	−2.16	.030	0.29 ± 0.05	5.84	.000

*Pres* Presence module, *Persp* Perspective module, *Affect* Affect module, *RCC* retest control cohort

### Self-Judgment (SCS)

The full-null model comparison was significant (*χ* ^2^ = 67.34, *df* = 13, *p* < 0.001, *p*_cor_ < 0.001, *R* ^2^_marg_ = 0.029, *R* ^2^_cond_ = 0.641, see Figs. 4 and 7). At T1, participants trained in either the Presence—although only significantly so in TC1—or the Affect module showed greater decreases in ratings of their *self-judgment* than the retest control group. At T2, the Affect module resulted in a marginally *smaller* decrease in ratings than the control group, whereas the decreases associated with the Affect module at T3 were *greater* than those in the RCC. Averaged over all three time points, the Affect modules led to greater decreases in ratings of *self-judgment* than the RCC. The changes in ratings after the Affect modules—averaged over the T1-T2 and T2-T3—did not significantly differ from the Perspective modules (see Table 5). Thus, although there is a general decrease over time and the ratings of the RCC also decrease between T1 and T2, it seems that overall especially the Affect module (in two of the three cohorts), but also the Presence module (in one of the two cohorts), were effective in decreasing self-judgments, whereas the Perspective module did not lead to any significant decreases compared to the other cohorts.

### Common Humanity (SCS)

In the full-null model comparison, the full model predicted the change in ratings on the *common humanity* scale of the SCS significantly better than the null model (*χ* ^2^ = 95.98, *df* = 13, *p* < 0.001, *p*_cor_ < 0.001, *R* ^2^_marg_ = 0.044, *R* ^2^_cond_ = 0.642; see Figs. 4 and 7). The contrasts revealed that neither the Presence nor the Perspective modules led to significantly different changes in ratings than the retest control group, but the Affect module led to consistent increases in ratings of *common humanity* compared to all other cohorts (see Table 5). These increases were of medium size between T0 and T1 as well as T1 and T2, as compared to the RCC (see Table 9).

### Isolation (SCS)

The full-null model comparison revealed that the training modules at the different time points differentially predicted the changes in ratings (*χ* ^2^ = 76.19, *df* = 13, *p* < 0.001, *p*_cor_ < 0.001, *R* ^2^_marg_ = 0.026, *R* ^2^_cond_ = 0.676; see Table 4 and Figs. 4 and 7). However, contrasts (see Table 6) revealed only two marginally significant comparisons—between Affect and Perspective at T2 as well as between Affect and the RCC over all three time points—which indicates that the significant full-null model comparison is likely driven by a main effect of time point, e.g., a general retest effect.

**Table 6:** Results of pairwise comparison contrasts derived from the linear mixed model of the effects of the different trainings and time points on the change scores of the subscales of the Self-Compassion Scale

Contrast	Isolation			Mindfulness			Overidentification		
	*β* ± SE	*Z*	*p*	*β* ± SE	*Z*	*p*	*β* ± SE	*Z*	*p*
Change between T0 and T1									
Affect-RCC	-0.11 ± 0.09	−1.13	.257	0.29 ± 0.09	3.33	.001	−0.07 ± 0.08	−0.87	.386
Pres (both)-RCC	-0.12 ± 0.08	−1.49	.136	0.12 ± 0.07	1.60	.110	−0.06 ± 0.07	−0.86	.389
Pres (TC1)-RCC	-0.14 ± 0.10	−1.47	.141	0.08 ± 0.09	0.95	.343	−0.13 ± 0.09	−1.56	.120
Pres (TC2)-RCC	-0.10 ± 0.09	−1.08	.282	0.15 ± 0.09	1.80	.072	0.01 ± 0.08	0.10	.922
Pres (both)-Affect	−0.01 ± 0.08	−0.17	.864	−0.17 ± 0.08	−2.19	.029	0.01 ± 0.08	0.14	.890
Pres (TC1)-Affect	−0.03 ± 0.10	−0.35	.727	−0.20 ± 0.09	−2.27	.023	−0.06 ± 0.09	−0.69	.492
Pres (TC2)-Affect	0.01 ± 0.10	0.06	.954	−0.13 ± 0.09	−1.50	.133	0.08 ± 0.09	0.94	.347
Change between T1 and T2									
Affect-RCC	-0.10 ± 0.10	−1.05	.294	0.27 ± 0.09	3.03	.002	−0.07 ± 0.09	−0.80	.425
Persp-RCC	0.07 ± 0.10	0.75	.453	0.13 ± 0.09	1.51	.132	−0.11 ± 0.09	−1.25	.213
Persp-Affect	0.17 ± 0.10	1.77	.077	−0.14 ± 0.09	−1.52	.129	−0.04 ± 0.09	−0.43	.667
Change between T2 and T3									
Affect-RCC	-0.05 ± 0.10	−0.48	.635	0.10 ± 0.09	1.19	.234	0.01 ± 0.09	0.16	.875
Persp-RCC	0.01 ± 0.10	0.15	.885	0.02 ± 0.09	0.17	.865	−0.14 ± 0.09	−1.63	.104
Persp-Affect	0.06 ± 0.10	0.61	.540	−0.09 ± 0.09	−1.01	.315	−0.15 ± 0.09	−1.77	.076
Change between T1 and T3									
Affect-RCC	-0.07 ± 0.06	−1.21	.228	0.19 ± 0.06	3.39	.001	−0.03 ± 0.06	−0.50	.621
Persp-RCC	0.04 ± 0.06	0.70	.481	0.07 ± 0.05	1.34	.181	−0.12 ± 0.06	−2.22	.026
Persp-Affect	0.12 ± 0.08	1.43	.154	−0.11 ± 0.08	−1.49	.137	−0.10 ± 0.07	−1.36	.173
Change between T0 and T3									
Affect-RCC	-0.08 ± 0.05	−1.81	.070	0.22 ± 0.04	5.22	.000	−0.04 ± 0.04	−1.00	.320

*Pres* Presence module, *Persp* Perspective module, *Affect* Affect module, *RCC* retest control cohort

### Mindfulness (SCS)

The full-null model comparison was significant (*χ* ^2^ = 113.47, *df* = 13, *p* < 0.001, *p*_cor_ < 0.001, *R* ^2^_marg_ = 0.061, *R* ^2^_cond_ = 0.616; see Figs. 4 and 7). The Presence module only in TC2 led to marginally greater change in ratings than the control condition. In general, the change after Affect modules was greater than the change in the participants in the RCC, although this difference was not significant between T2 and T3 (but the effect was of medium size at the other time points, see Table 10). The Perspective module did not differ significantly from the RCC and the Affect module (see Table 6 for contrasts). Thus, *mindfulness* as part of self-compassion benefitted from specific training in the Affect modules.

### Overidentification (SCS)

The full-null model comparison was significant (*χ* ^2^ = 94.66, *df* = 13, *p* < 0.001, *p*_cor_ < 0.001, *R* ^2^_marg_ = 0.041, *R* ^2^_cond_ = 0.582; see Figs. 4 and 7). The decreases in ratings averaged both Perspective modules were significantly greater than those in the RCC, but this effect was not significant at the separate time points (see Table 6). Because this is the only significant contrast, the significance of the full-null model comparison is thus likely driven by a retest effect.

### Kindness (CS)

**Fig. 5:**
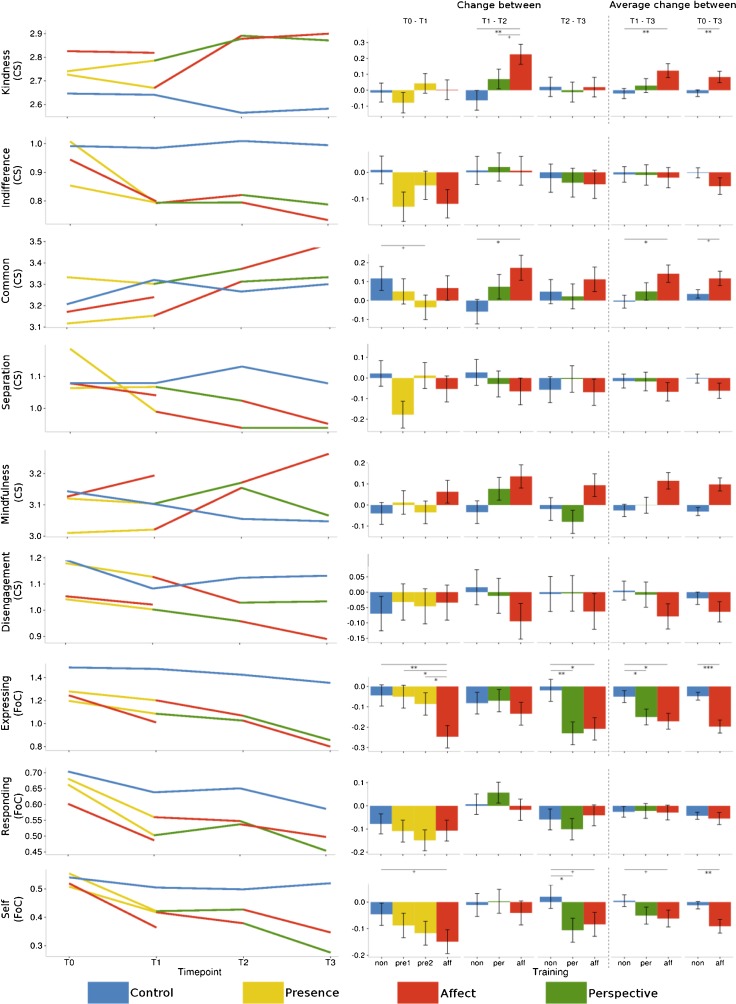
Averaged raw data per cohort and time point (*left*) and estimates and standard errors of the changes between time points derived from the contrasts of the linear mixed models (*right*) per subscale of the *Compassion Scale* and the *Fear of Compassion* Scale. The three *leftmost bar charts* represent the change between two subsequent time points, whereas the two *right bar chart columns* represent the average change for both Perspective modules (plus the matching control and Affect modules) and the average change for all three Affect modules (plus the matching control cohorts). +*p* < .1; *p ≤ .05; **p ≤ .01; ***p ≤ .001

The full-null model comparison was significant (*χ* ^2^ = 33.15, *df* = 13, *p* = 0.002, *p*_cor_ = 0.008, *R* ^2^_marg_ = 0.032, *R* ^2^_cond_ = 0.586; see Table 7 for contrasts). The Affect module, averaged over all time points, led to greater increases than the RCC, but this effect was driven by the steep increase in ratings of medium effect size between T1 and T2 (see Figs. 5 and 8 and Table 10).

**Table 7:**
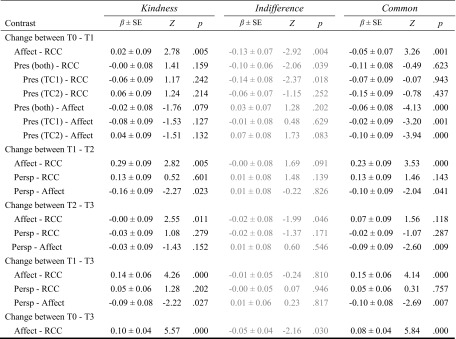
Results of pairwise comparison contrasts derived from the linear mixed model of the effects of the different trainings and time points on the change scores of the subscales of the Compassion Scale

Gray numbers indicate nonsignificant likelihood ratio tests for the full model

*Pres* Presence module, *Persp* Perspective module, *Affect* Affect module, *RCC* retest control cohort

### Indifference (CS)

The full-null model comparison was not significant (*χ* ^2^ = 20.08, *df* = 13, *p* = 0.093, *p*_cor_ = 0.214, *R* ^2^_marg_ = 0.17, *R* ^2^_cond_ = 0.606; see Figs. 5 and 8 and Table 7 for contrasts). Adding the time point and module information therefore does not explain the variance in the *indifference* ratings better than the subject-specific information included in the null model.

### Common (CS)

The full-null model comparison was significant (*χ* ^2^ = 35.08, *df* = 13, *p* < 0.001, *p*_cor_ = 0.005, *R* ^2^_marg_ = 0.025, *R* ^2^_cond_ = 0.502; see Figs. 5 and 8 and Table 7 for contrasts). The contrasts revealed that the comparison between the overall Affect module change and that in the RCC is marginally significant, which is mainly driven by a significant difference in the change between T1 and T2. Therefore, the Affect module led to slight increases above and beyond the general retest effect.

### Separation (CS)

The full-null model comparison was not significant (*χ* ^2^ = 21.08, *df* = 13, *p* = 0.071, *p*_cor_ = 0.214, *R* ^2^_marg_ = 0.012, *R* ^2^_cond_ = 0.547; see Figs. 5 and 8 and Table 8 for contrasts).

**Table 8:**
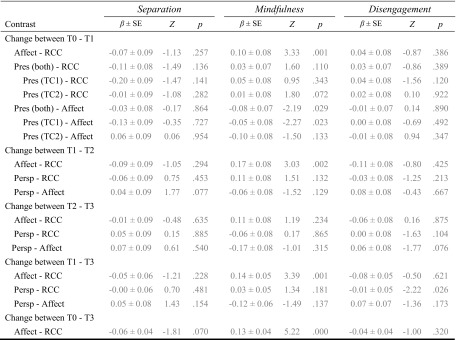
Results of pairwise comparison contrasts derived from the linear mixed model of the effects of the different trainings and time points on the change scores of the subscales of the Compassion Scale

Gray numbers indicate nonsignificant likelihood ratio tests for the full model

*Pres* Presence module, *Persp* Perspective module, *Affect* Affect module, *RCC* retest control cohort

### Mindfulness (CS)

The full-null model comparison was significant, but only if uncorrected for multiple testing (*χ* ^2^ = 23.62, *df* = 13, *p* = 0.035, *p*_cor_ = 0.139, *R* ^2^_marg_ = 0.015, *R* ^2^_cond_ = 0.517; see Figs. 5 and 8 and Table 8 for contrasts), which seems to be due to consistent increases in ratings after the Affect modules.

### Disengagement (CS)

The full-null model comparison was not significant (*χ* ^2^ = 15.46, *df* = 13, *p* = 0.280, *p*_cor_ = 0.280, *R* ^2^_marg_ = 0.014, *R* ^2^_cond_ = 0.580; see Fig. 5 and Table 8 for contrasts).

### Expressing (FoC)

The full-null model comparison was significant (*χ* ^2^ = 128.50, *df* = 13, *p* < 0.001, *p*_cor_ < 0.001, *R* ^2^_marg_ = 0.089, *R* ^2^_cond_ = 0.728). The contrasts revealed that participants after the Affect and Perspective modules—not between T1 and T2 though—showed greater decreases in ratings than the control group, but the Presence module did not differ from the control group (see Figs. 5 and 9 and Table 9 for contrasts).

**Table 9:** Results of pairwise comparison contrasts derived from the linear mixed model of the effects of the different trainings and time points on the change scores of the subscales of the Fear of Compassion scale

Contrast	Expressing			Responding			Self		
	*β* ± SE	*Z*	*p*	*β* ± SE	*Z*	*p*	*β* ± SE	*Z*	*p*
Change between T0 and T1									
Affect-RCC	-0.20 ± 0.08	−2.70	.007	−0.03 ± 0.06	−0.47	.637	−0.10 ± 0.06	−1.68	.093
Pres (both)-RCC	-0.02 ± 0.07	−0.37	.711	−0.05 ± 0.05	−0.96	.337	−0.06 ± 0.05	−1.07	.284
Pres (TC1)-RCC	-0.01 ± 0.08	−0.08	.939	−0.03 ± 0.06	−0.50	.618	−0.04 ± 0.06	−0.68	.498
Pres (TC2)-RCC	-0.04 ± 0.08	−0.56	.575	−0.07 ± 0.06	−1.15	.250	−0.07 ± 0.06	−1.16	.245
Pres (both)-Affect	0.18 ± 0.07	2.66	.008	−0.02 ± 0.06	−0.40	.690	0.05 ± 0.06	0.84	.399
Pres (TC1)-Affect	0.20 ± 0.08	2.51	.012	−0.00 ± 0.07	−0.03	.973	0.06 ± 0.06	0.95	.343
Pres (TC2)-Affect	0.16 ± 0.08	2.08	.038	−0.04 ± 0.06	−0.66	.508	0.03 ± 0.06	0.50	.615
Change between T1 and T2									
Affect-RCC	-0.05 ± 0.08	−0.69	.494	−0.02 ± 0.06	−0.37	.713	−0.03 ± 0.06	−0.48	.634
Persp-RCC	0.01 ± 0.08	0.16	.873	0.05 ± 0.06	0.80	.424	0.01 ± 0.06	0.23	.820
Persp-Affect	0.07 ± 0.08	0.83	.405	0.07 ± 0.06	1.14	.253	0.04 ± 0.06	0.69	.489
Change between T2 and T3									
Affect-RCC	-0.19 ± 0.08	−2.47	.014	0.02 ± 0.06	0.27	.785	−0.10 ± 0.06	−1.66	.097
Persp-RCC	-0.21 ± 0.08	−2.73	.006	−0.04 ± 0.06	−0.65	.513	−0.13 ± 0.06	−1.99	.046
Persp-Affect	−0.02 ± 0.08	−0.28	.782	−0.06 ± 0.06	−0.92	.358	−0.02 ± 0.06	−0.34	.731
Change between T1 and T3									
Affect-RCC	-0.12 ± 0.05	−2.51	.012	−0.00 ± 0.04	−0.08	.939	−0.07 ± 0.04	−1.74	.082
Persp-RCC	-0.10 ± 0.05	−2.06	.040	0.00 ± 0.04	0.11	.914	−0.06 ± 0.04	−1.46	.146
Persp-Affect	0.02 ± 0.07	0.33	.740	0.01 ± 0.06	0.13	.895	0.01 ± 0.06	0.20	.840
Change between T0 and T3									
Affect-RCC	-0.15 ± 0.04	−4.00	.000	−0.01 ± 0.03	−0.39	.698	−0.08 ± 0.03	−2.67	.008

*Pres* Presence module, *Persp* Perspective module, *Affect* Affect module, *RCC* retest control cohort

### Responding (FoC)

The full-null model comparison was significant (*χ* ^2^ = 49.60, *df* = 13, *p* < 0.001, *p*_cor_ < 0.001, *R* ^2^_marg_ = 0.020, *R* ^2^_cond_ = 0.691; see Figs. 5 and 9 and Table 9 for contrasts). However, none of the contrasts reached significance, which indicates that this effect is likely due to a main effect of time point, e.g., a general retest effect.

### Self (FoC)

The full-null model comparison was significant (*χ* ^2^ = 60.93, *df* = 13, *p* < 0.001, *p*_cor_ < 0.001, *R* ^2^_marg_ = 0.030, *R* ^2^_cond_ = 0.669; see Figs. 5 and 9 and Table 9 for contrasts). The contrasts revealed that decreases in ratings after the Affect modules at T1 and T3 were marginally greater than in the RCC, which results in a significant difference when averaged over all three time points. At T3, the Perspective module also led to greater decreases in ratings than the RCC. Thus, it seems that the Affect module leads to slight decreases in fear of self-compassion, which are further enhanced in the Perspective module if it follows the Affect module (Table 10).

**Table 10:**
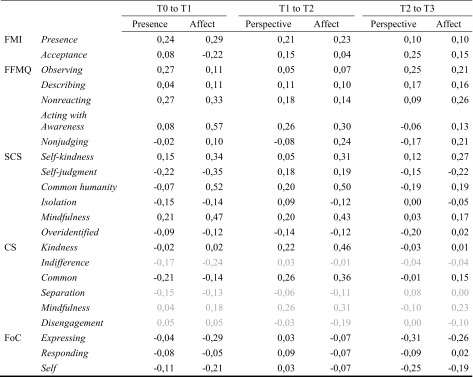
Effect sizes per module and time point calculated as described in Morris (2008) for Pretest-Posttest-Control Group Designs for all subscales

Gray numbers indicate nonsignificant likelihood ratio tests for the full model

## Discussion

The aim of this study was to examine the specific effects of different meditation-based mental training practices on mindfulness, compassion, and self-compassion trait questionnaires. We were specifically interested in testing whether present-moment- and attention-focused practices are sufficient to elicit changes in a variety of facets of mindfulness and compassion (the “mindfulness cascade” model, as indicated by, e.g., Grossman 2008) or whether acceptance- and compassion-focused facets require specific cultivation through explicit socio-affective compassion-based practices (Desbordes et al. 2015; Neff and Dahm 2015). To test these assumptions, we used data that was collected in the context of the ReSource Project, a 9-month longitudinal study in which participants sequentially completed three 3-month modules focused on present-moment awareness and attention (Presence), socio-affective and motivational skills (Affect), and meta-/socio-cognitive skills (Perspective). To get a nuanced measurement space of different aspects of mindfulness and compassion, we assessed self-reported mindfulness with the FMI (Buchheld et al. 2001) and the FFMQ (Baer et al. 2006), and (self-)compassion with the SCS (Neff 2003), the CS (Pommier 2011), and the FoC (Gilbert et al. 2011).

The pattern of results revealed that the three different types of mental training modules implemented in the ReSource Project had both broad and specific effects on the mindfulness and compassion-related self-report scales. Importantly, the Presence module did *not* lead to broad effects across a variety of subscales such as nonjudgment, acceptance, and all (self-)compassion facets. In contrast, ratings on these subscales were specifically increased after the socio-affective compassion-based Affect module and—to a lesser extent—the Perspective module.

With respect to the mindfulness scales, the present-moment- and attention-focused Presence module significantly increased, as expected, ratings on the *observing*, *nonreacting* (both FFMQ), and *presence* (FMI) subscales. These effects were not unique to the Presence module but were further cultivated by the more intersubjective Affect and/or Perspective modules. The Perspective module resulted in changes in *acceptance* (FMI), *observing*, and slight increases in *describing* (both FFMQ). Most importantly, the compassion-based Affect module had the broadest effects, leading to increases on a number of facets of mindfulness: *presence* (FMI), *observing*, *describing*, *nonreacting*, *acting with awareness*, and *nonjudging* (all FFMQ). The increase of *nonjudging* (FFMQ) ratings was especially unique, as the ratings on this subscale were rather decreased after the Perspective module.

Regarding self-compassion and compassion, ratings on the positive subscales of the SCS—*self-kindness*, *common humanity*, and *mindfulness—*were clearly and uniquely increased through the Affect module. The Affect module also influenced one (but only one) of the negatively phrased subscales, *self-judgment*, which was also decreased in one of the cohorts who had completed the Presence modules. Ratings of *isolation* and *overidentification* decreased over time but not specifically due to the trainings. Ratings on the CS were similarly affected by the Affect module only. Again, (marginally) significant changes in ratings after the Affect module were only found on the three subscales that contain positively phrased items—*kindness*, *common*, and *mindfulness*—but not their negative counterparts. Finally, out of the three FoC subscales, ratings of *expressing* compassion when facing the suffering of others were significantly decreased after both the Affect and the Perspective modules, and ratings of fear of self-compassion were reduced after the Affect module only.

These results indicate that even 3 months of practicing present-moment-focused attention-based practices, as implemented in the Presence module, is not sufficient to induce broad changes across all facets of self-rated mindfulness, compassion, and self-compassion as implied in cascade-like views of mindfulness. Rather, some facets of mindfulness included in the two mindfulness questionnaires assessed here, that is *acceptance*, *nonjudging*, and—surprisingly—*acting with awareness*, benefitted from specific cultivation in the Perspective or the Affect modules. In addition, changes in compassion and self-compassion were elicited almost exclusively by the Affect module. Therefore, the results do not support the cascade model of mindfulness, at least not after 3 months of training in each module as implemented in the ReSource project.

**Fig. 6:**
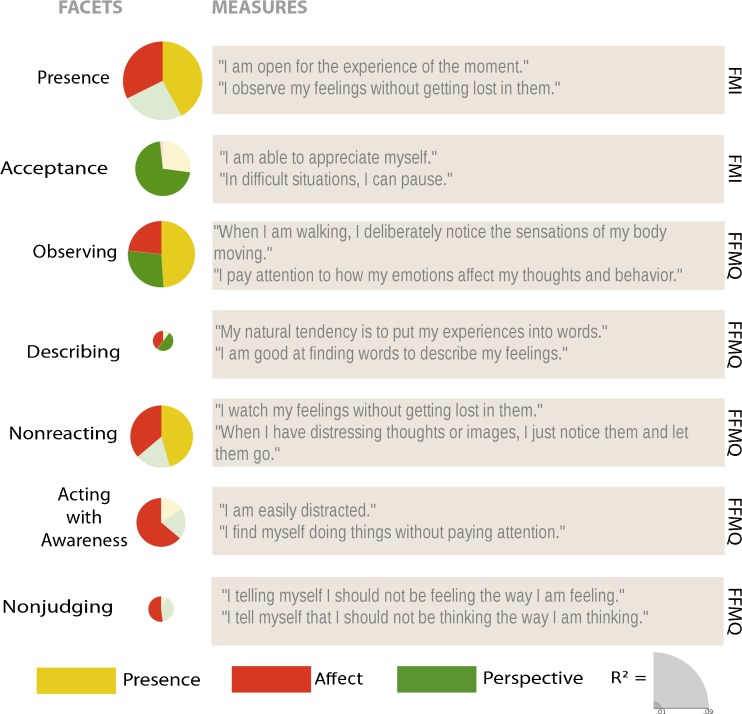
Visual summary and example questions of the results per subscale of the *Freiburg Mindfulness Inventory* and the *Five-Facet Mindfulness Questionnaire*. *Pie charts* are scaled by the marginal *R* ^2^, and *slices* represent the *β* values of the averaged contrasts. Nonsignificant results are transparent

Interestingly, *acceptance* and *nonjudging*, as measured by the FMI and FFMQ respectively, seem to be distinguishable capacities targeted by different meditation-based mental practices, the former by the Perspective and the latter by the Affect module. A close examination of the items (see Fig. 6 for further examples) on these scales indicated that *acceptance* (FMI) rather involves both a global form of self-compassion (e.g., “I am able to appreciate myself”) but importantly also responses related to inner balance and equanimity (e.g., “I experience moments of inner peace and ease, even when things get hectic and stressful” or “In difficult situations, I can pause”). Thus, the *acceptance* subscale of the FFMQ seems to measure acceptance as rather related to aspects of equanimity, nonreactivity, and meta-cognitive awareness. Cultivating such capacities was indeed the focus of the Perspective module wherein participants learned to watch their thoughts without elaborating, or to watch aspects of themselves without identifying with them. In contrast, *nonjudging* as conceptualized in the FFMQ involves questions related to specific self-judgment and self-criticism (e.g., “I criticize myself for having irrational or inappropriate feelings,” “I tell myself I should not be feeling the way I am feeling”). The Affect module involved constructive meditation types (Dahl et al. 2015) in that it taught participants how to activate positive feelings of benevolence, care, and kindness to themselves and others. Thus, it is not surprising to have observed that scales assessing the tendency to be less judgmental and critical with oneself improved after the Affect module.

A number of unexpected results emerged. Firstly, the Affect but not the Presence module increased *acting with awareness*. We did not predict this finding, as the *acting with awareness* subscale clearly measures attention-related skills, which is the primary focus of the Presence module. However, basic loving-kindness practice also (a) helps to increase your attentional capacities as you have to hold inner imagery and intentions in mind over long periods of practice and (b) includes a very strong motivation and action component, focusing your attention on acting for the benefit of others. Furthermore, all items of the *acting with awareness* subscale were negatively formulated (and subsequently recoded). It could therefore be the case that the self-compassion taught in the Affect module made participants less judgmental of their wandering mind or everyday attention slips and that they consequently rated themselves lower, i.e., more positively, on these negative items. Similarly, the diverging findings of the *nonjudging* and *acceptance* subscales mentioned above may have been driven by the initially negative formulation (and subsequent recoding) of the *nonjudging* subscale’s items. Taken together, these interpretations thus suggest that the valence of self-report items may strongly influence how much they were endorsed after the different modules. In other words, becoming more self-compassionate after the Affect module possibly leads to less agreement to negative, self-judgmental characteristics. In contrast, realizing after the Perspective module that there are always different—including negative—inner parts and perspectives might lead to embracing negative traits more.

**Fig. 7:**
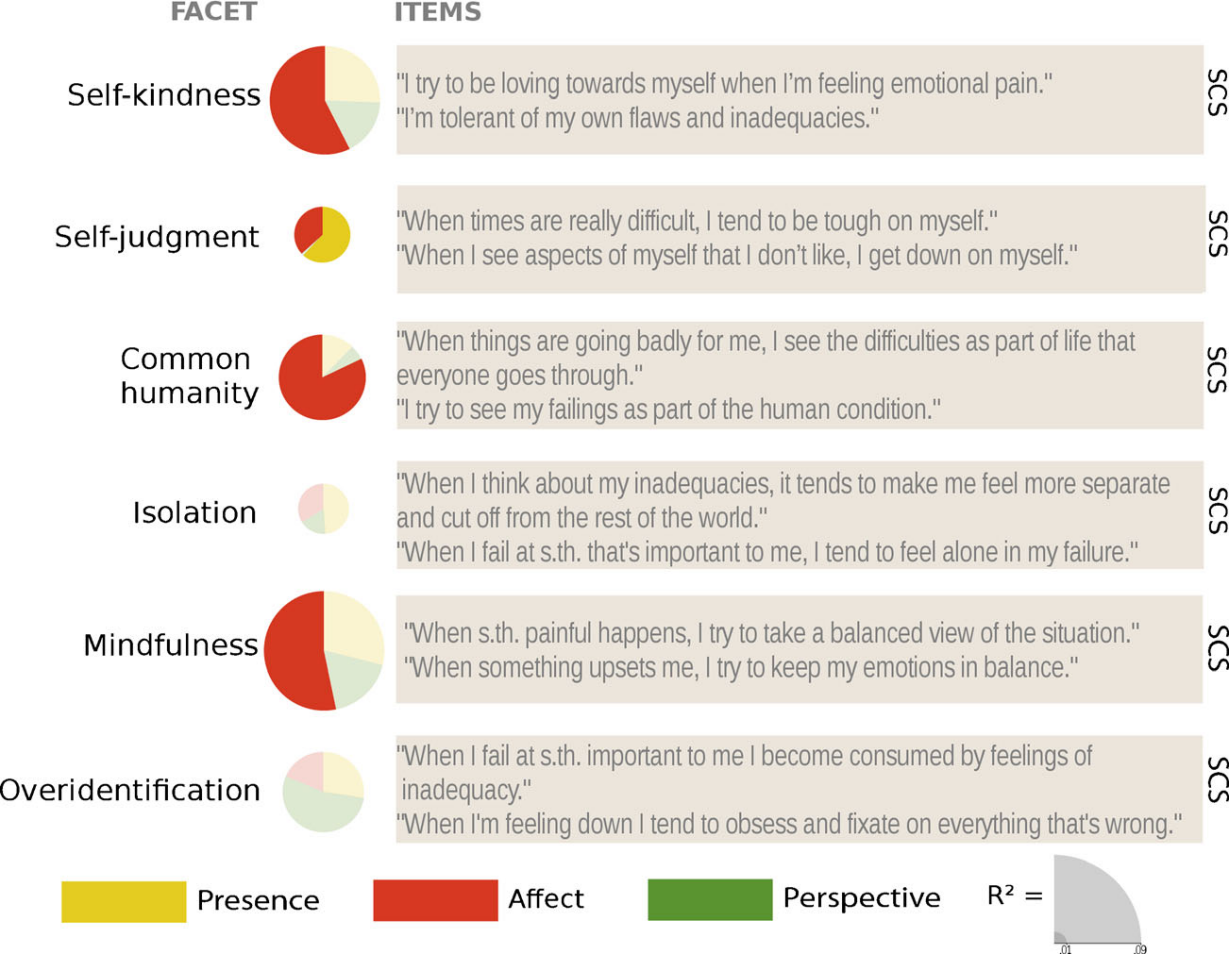
Visual summary and example questions of the results per subscale of the *Self-Compassion Scale*. *Pie charts* are scaled by the marginal *R* ^2^, and *slices* represent the *β* values of the averaged contrasts. Nonsignificant results are transparent

**Fig. 8:**
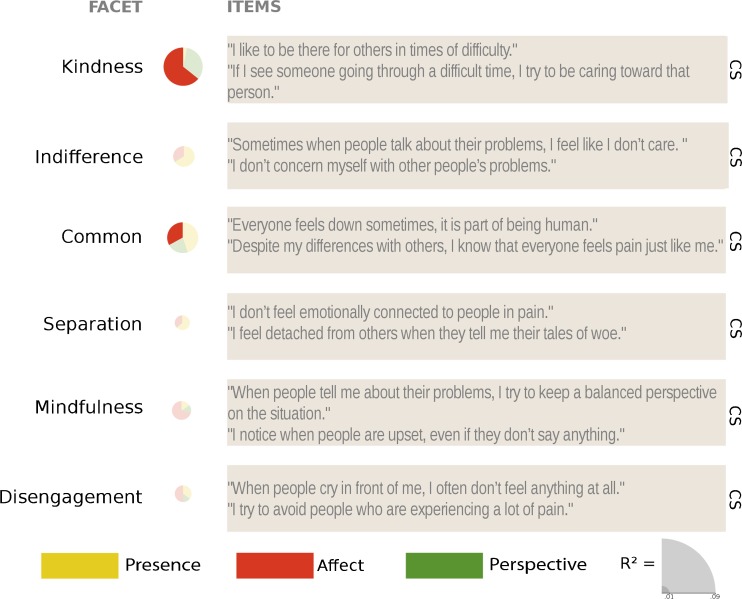
Visual summary and example questions of the results per subscale of *Compassion Scale*. *Pie charts* are scaled by the marginal *R* ^2^, and *slices* represent the *β* values of the averaged contrasts. Nonsignificant results are transparent

**Fig. 9:**
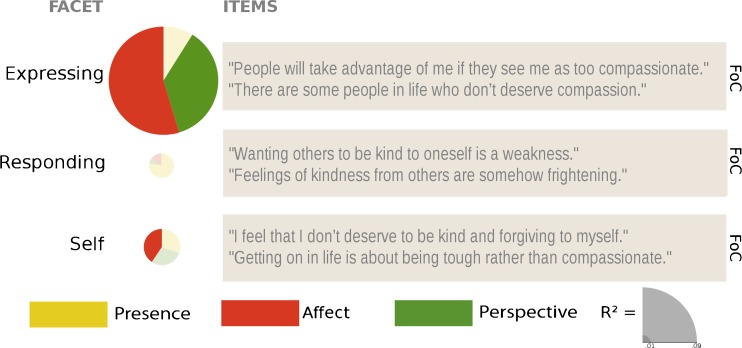
Visual summary and example questions of the results per subscale of the *Fear of Compassion* scale. *Pie charts* are scaled by the marginal *R* ^2^, and *slices* represent the *β* values of the averaged contrasts. Nonsignificant results are transparent

The findings presented here have a number of other important implications for the understanding of, and the research on, mindfulness and compassion. Although all three modules generally increased many facets of self-rated mindfulness, the size of the changes over the 9 months differed between facets. In general, *R* ^2^-like effect sizes (see sizes of pie charts in Figs. 6, 7, 8, and 9) of most of the mindfulness subscales (especially the ones who already benefitted from the Presence module), the positive self-compassion subscales, and the subscale measuring fear of *expressing* compassion were among the largest. Importantly, the socio-affective compassion-based Affect training module from the ReSource Project had the broadest effect across most self-report subscales of the mindfulness, compassion and self-compassion questionnaires. This has two important implications: firstly, it indicates that acceptance and (self-)compassion did not automatically emerge from the cultivation of present-moment and attention-based meditation practices as implemented in 3-month Presence training modules, but required explicit loving-kindness, gratitude, and compassion-based practices possibly activating affiliative and care-based motivational systems (Klimecki et al. 2014; Singer and Klimecki 2014). Secondly, and possibly surprisingly, the Affect module also influenced basic present-moment- and attention-focused mindfulness facets, such as acting with awareness and presence, which were expected to be most closely related to practices implemented in the Presence module. Therefore, if there is not a cascade from mindfulness to compassion, there might be a cascade of beneficial effects from compassion to mindfulness. One explanation might be that, although we tried to isolate the capacities trained, the socio-affective practices in the Affect module also strongly depend on present-moment-focused attention-, and interoception-related skills. Accordingly, each practice, whether it was the affect dyad or Loving-kindness meditation, always started with rooting oneself in owns own body and the present moment and required concentration of an object of attention, such as an inner image or another person. This is in line with the Buddhist conception that basic attention exercises and mindfulness build the foundation for other meditation practices.

To summarize the results, we can return to Kabat-Zinn’s (1994) influential definition of mindfulness as consisting of (1) attention to the present moment (2) in a nonjudgmental way. In line with existing literature, we showed that the first component of that definition is increased by 3 months of present-moment- and attention-focused exercises. This is in line with classical views of basic attention-focused mindfulness that specifically exclude secondary processes (Rapgay and Bystrisky 2009). We also demonstrated that the second part of that definition, the more controversial elements of acceptance and nonjudgmental awareness (Bishop et al. 2004; Grossman and Van Dam 2011), likely requires targeted and explicit cultivation of these qualities. These findings imply that meditation-based interventions might be especially effective to increase a variety of qualities if they include explicit practices focused on care, benevolence, acceptance, and/or nonjudgment. This is in line with earlier work suggesting that equanimity and self-compassion appear to be mechanisms through which mindfulness-based practices lead to positive health outcomes and increased well-being (e.g., Birnie et al. 2010; Desbordes et al. 2015; Gu et al. 2015; Keng et al. 2012; Szekeres and Wertheim 2015; Woodruff et al. 2014), but also with the work showing that mindfulness- and compassion-based interventions lead to increased well-being via different pathways (Desbordes et al. 2012). Note, however, that in most 8-week mindfulness-based programs such as MBSR, the implicit inclusion of compassion and acceptance in the practice instructions may explain previous findings of increases in compassion and/or self-compassion after such interventions (Birnie et al. 2010; Gu et al. 2015; Keng et al. 2012). Nevertheless, explicitly cultivating compassion and self-compassion will likely boost these positive outcomes (Desbordes et al. 2015; Hofmann et al. 2011; Neff and Dahm 2015). The results presented here support the notion that “mindfulness and compassion are complementary practices and can work in mutually reinforcing ways” (Germer and Barnhofer 2017) and warn against overgeneralized claims that, for example, breath-focused meditation practices alone will necessarily bring about changes in compassion, cooperation or other ethical behaviors.

### Limitations and Suggestions for Further Research

The current study has some limitations. The questionnaires included in this study also only represent a subset of available (mindfulness) questionnaires, which are in general rather contested. Moreover, self-report questionnaire measures must generally be treated with some caution. Participants in the ReSource Project committed to a daily meditation practice and extensive psychological and neuroscientific testing; within this context, it is possible that demand characteristics influenced self-reports. Of course, it is impossible to conduct an intervention like this as a double-blind study, so participants knew what they learned and the questionnaires reported here measure characteristics that are closely related to the practices.

Secondly, each of the modules in the ReSource Project included a range of both novel and established mental training exercises. While these exercises were chosen to target specific mental capacities, the unique combinations may lead to difficulties generalizing from this study to other already established meditation-based mental training interventions. Therefore, further research focusing on the differential effects of single specific exercises would further help specifying the specific mechanisms driving observed changes after mental training.

To conclude, our findings of differential effects of different types of contemplative practices on the array of mindfulness and compassion scales widely used in the field warn against the notion that simple trainings aimed at the optimization of attention skills alone will have far-reaching consequences. In contrast, the results indicate that we should conduct research with a fine-grained view of contemplative mental training that includes additional focus on of the ethical and affective qualities related to compassion and self-acceptance, qualities not only important for the individual health but also for overall flourishing in terms of global cooperation and responsibility.

## Electronic supplementary material

ESM 1 (DOCX 262 kb)

## References

[CR1] Baayen RH (2008). Analyzing linguistic data: a practical introduction to statistics using R.

[CR2] Baer RA (2003). Mindfulness training as a clinical intervention: a conceptual and empirical review. Clinical Psychology: Science and Practice.

[CR3] Baer RA, Smith GT, Hopkins J, Krietemeyer J, Toney L (2006). Using self-report assessment methods to explore facets of mindfulness. Assessment.

[CR4] Baer, R. A., Smith, G. T., Lykins, E., Button, D., Krietemeyer, J., Sauer, S., … Williams, J. M. G. (2008). Construct validity of the five facet mindfulness questionnaire in meditating and nonmeditating samples. *Assessment*, *15*(3), 329–42. doi:10.1177/1073191107313003 10.1177/1073191107313003 18310597

[CR5] Bergomi C, Tschacher W, Kupper Z (2013). The assessment of mindfulness with self-report measures: existing scales and open issues. Mindfulness.

[CR6] Birnie K, Speca M, Carlson LE (2010). Exploring self-compassion and empathy in the context of mindfulness-based stress reduction (MBSR). Stress and Health.

[CR7] Bishop, S. R., Lau, M., Shapiro, S., Carlson, L., Anderson, N D., Carmody, J., … Devins, G. (2004). Mindfulness: a proposed operational definition. *Clinical Psychology: Science and Practice*, *11*(3), 230–241. doi:10.1093/clipsy/bph077

[CR8] Bodhi B (2011). What does mindfulness really mean? A canonical perspective. Contemporary Buddhism.

[CR9] Brown KW, Ryan RM (2003). The benefits of being present: mindfulness and its role in psychological well-being. Journal of Personality and Social Psychology.

[CR10] Brown KW, Ryan RM (2004). Perils and promise in defining and measuring mindfulness: observations from experience. Clinical Psychology.

[CR11] Buchheld N, Grossman P, Walach H (2001). Measuring mindfulness in insight meditation (Vipassana) and meditation-based psychotherapy: the development of the Freiburg Mindfulness Inventory (FMI). Journal of Meditation and Meditation Research.

[CR12] Chiesa A (2013). The difficulty of defining mindfulness: current thought and critical issues. Mindfulness.

[CR13] Dahl, C. J., Lutz, A., Davidson, R. J. (2015). Reconstructing and deconstructing the self: cognitive mechanisms in meditation practice. *Trends in Cognitive Sciences, 19*(9), 515–523. 10.1016/j.tics.2015.07.001 PMC4595910 26231761

[CR14] Davis, M. H. (1983). Measuring individual differences in empathy: Evidence for a multidimensional approach. *Journal of Personality and Social Psychology, 44*(1), 113–126.

[CR15] Core Team R (2016). R: a language and environment for statistical computing.

[CR16] Desbordes G, Negi LT, Pace TWW, Wallace BA, Raison CL, Schwartz EL (2012). Effects of mindful-attention and compassion meditation training on amygdala response to emotional stimuli in an ordinary, non-meditative state. Frontiers in Human Neuroscience.

[CR17] Desbordes, G., Gard, T., Hoge, E. A., Hölzel, B. K., Kerr, C., Lazar, S. W., … Vago, D. R. (2015). Moving beyond mindfulness: defining equanimity as an outcome measure in meditation and contemplative research. *Mindfulness*, *6*(2), 356–372. doi:10.1007/s12671-013-0269-8 10.1007/s12671-013-0269-8 PMC4350240 25750687

[CR18] Dobson AJ, Barnett A (2008). An introduction to generalized linear models.

[CR19] Dreyfus G (2011). Is mindfulness present-centred and non-judgmental? A discussion of the cognitive dimensions of mindfulness. Contemporary Buddhism.

[CR20] Eberth J, Sedlmeier P (2012). The effects of mindfulness meditation: a meta-analysis. Mindfulness.

[CR21] Forstmeier W, Schielzeth H (2011). Cryptic multiple hypotheses testing in linear models: Overestimated effect sizes and the winner’s curse. Behavioral Ecology and Sociobiology.

[CR22] Galante J, Galante I, Bekkers M-J, Gallacher J (2014). Effect of kindness-based meditation on health and well-being: a systematic review and meta-analysis. Journal of Consulting and Clinical Psychology.

[CR23] Germer C, Barnhofer T, Gilbert P (2017). Mindfulness and compassion: similarities and differences. Compassion: concepts, research and applications.

[CR24] Gethin R (1998). The foundations of Buddhism.

[CR25] Gilbert P, McEwan K, Matos M, Rivis A (2011). Fears of compassion: development of three self-report measures. Psychology and Psychotherapy.

[CR26] Goetz J, Keltner D, Simon-Thomas E (2010). Compassion: an evolutionary analysis and empirical review. Psychological Bulletin.

[CR27] Grossman P (2008). On measuring mindfulness in psychosomatic and psychological research. Journal of Psychosomatic Research.

[CR28] Grossman P, Van Dam NT (2011). Mindfulness, by any other name…: trials and tribulations of sati in western psychology and science. Contemporary Buddhism.

[CR29] Grossman P, Niemann L, Schmidt S, Walach H (2004). Mindfulness-based stress reduction and health benefits: a meta-analysis. Journal of Psychosomatic Research.

[CR30] Gu J, Strauss C, Bond R, Cavanagh K (2015). How do mindfulness-based cognitive therapy and mindfulness-based stress reduction improve mental health and wellbeing? A systematic review and meta-analysis of mediation studies. Clinical Psychology Review.

[CR31] Hanley AW, Abell N, Osborn DS, Roehrig AD, Canto AI (2016). Mind the gaps: are conclusions about mindfulness entirely conclusive?. Journal of Counseling and Development.

[CR32] Hofmann SG, Grossman P, Hinton DE (2011). Loving-kindness and compassion meditation: potential for psychological interventions. Clinical Psychology Review.

[CR33] Holm S (1979). A simple sequentially rejective multiple test procedure. Scandinavian Journal of Statistics.

[CR34] Hothorn T, Bretz F, Westfall P (2008). Simultaneous inference in general parametric models. Biometrical Journal.

[CR35] Jazaieri, H., Jinpa, G. T., McGonigal, K., Rosenberg, E. L., Finkelstein, J., Simon-Thomas, E., … Goldin, P. R. (2013a). Enhancing compassion: a randomized controlled trial of a compassion cultivation training program. *Journal of Happiness Studies*, *14*(4), 1113–1126. doi:10.1007/s10902-012-9373-z

[CR36] Jazaieri H, McGonigal K, Jinpa T, Doty JR, Gross JJ, Goldin PR (2013). A randomized controlled trial of compassion cultivation training: effects on mindfulness, affect, and emotion regulation. Motivation and Emotion.

[CR37] Kabat-Zinn J (1982). An outpatient program in behavioral medicine for chronic pain patientsbased on the practice of mindfulness meditation—theoretical considerations and preliminary results. General Hospital Psychiatry.

[CR38] Kabat-Zinn J (1994). Wherever you go, there you are: mindfulness meditation in everyday life.

[CR39] Kabat-Zinn J (2003). Mindfulness-based interventions in context: past, present, and future. Clinical Psychology: Science and Practice.

[CR40] Kanske P, Böckler A, Trautwein F-M, Singer T (2015). Dissecting the social brain: Introducing the EmpaToM to reveal distinct neural networks and brain-behavior relations for empathy and theory of mind. NeuroImage.

[CR41] Keng S-L, Smoski MJ, Robins CJ, Ekblad AG, Brantley JG (2012). Mechanisms of change in mindfulness-based stress reduction: self-compassion and mindfulness as mediators of intervention outcomes. Journal of Cognitive Psychotherapy.

[CR42] Klimecki OM, Leiberg S, Ricard M, Singer T (2014). Differential pattern of functional brain plasticity after compassion and empathy training. Social Cognitive and Affective Neuroscience.

[CR43] Kohls N, Sauer S, Walach H (2009). Facets of mindfulness—results of an online study investigating the Freiburg mindfulness inventory. Personality and Individual Differences.

[CR44] Lutz A, Dunne JD, Davidson RJ, Zelazo PD, Moscovitch M, Thompson E (2007). Meditation and the neuroscience of consciousness: an introduction. Cambridge handbook of consciousness.

[CR45] Lutz A, Slagter HA, Dunne JD, Davidson RJ (2008). Attention regulation and monitoring in meditation. Trends in Cognitive Sciences.

[CR46] Morris, S. B. (2008). Estimating effect sizes from pretest-posttest-control group designs. *Organizational Research Methods, 11*(2), 364-386.

[CR47] Nakagawa S, Schielzeth H (2013). A general and simple method for obtaining R2 from generalized linear mixed-effects models. Methods in Ecology and Evolution.

[CR48] Neff KD (2003). Development and validation of a scale to measure self-compassion. Self and Identity.

[CR49] Neff KD, Dahm KA, Robinson M, Meier B, Ostafin B (2015). Self-compassion: what it is, what it does, and how it relates to mindfulness. Handbook of mindfulness and self-regulation.

[CR50] Petersen SE, Posner MI (2012). The attention system of the human Brain: 20 years after. Annual Review of Neuroscience.

[CR51] Pinheiro, J. C., Bates, D., DebRoy, S., Sarkar, D., & R Core Team. (2016). *_nlme: linear and nonlinear mixed effects models*. Retrieved from http://cran.r-project.org/package=nlme.

[CR52] Pommier, E. A. (2011). The compassion scale. *Dissertation Abstracts International Secton A: Humanities and Social Sciences*, *72*(1174).

[CR53] Premack D, Woodruff G (1978). Does the chimpanzee have a theory of mind?. Behavioral and Brain Sciences.

[CR54] Raes F, Pommier E, Neff KD, Van Gucht D (2011). Construction and factorial validation of a short form of the Self-Compassion Scale. Clinical Psychology & Psychotherapy.

[CR55] Rapgay L, Bystrisky A (2009). Classical mindfulness: an introduction to its theory and practice for clinical application. Annals of the New York Academy of Sciences.

[CR56] Salzberg S (1995). Loving-kindness—the revolutionary art of happiness.

[CR57] Salzberg S (2011). Mindfulness and loving-kindness. Contemporary Buddhism.

[CR58] Sauer S, Walach H, Offenbächer M, Lynch S, Kohls N (2011). Measuring mindfulness: a rasch analysis of the Freiburg Mindfulness Inventory. Religions.

[CR59] Segal ZV, Williams JMG, Teasdale JD (2002). Mindfulness-based cognitive therapy for depression: a new approach to preventing relapse.

[CR60] Shapiro SL, Carlson LE, Astin JA, Freedman B (2006). Mechanisms of mindfulness. Journal of Clinical Psychology.

[CR61] Singer T (2012). The past, present and future of social neuroscience: a European perspective. NeuroImage.

[CR62] Singer T, Klimecki OM (2014). Empathy and compassion. Current Biology: CB.

[CR63] Singer T, Kok BE, Bornemann B, Zurborg S, Bolz M, Bochow CA (2016). The ReSource Project. Background, design, samples, and measurements.

[CR64] Szekeres RA, Wertheim EH (2015). Evaluation of vipassana meditation course effects on subjective stress, well-being, self-kindness and mindfulness in a community sample: Post-course and 6-month outcomes. Stress and Health.

[CR65] Vago DR, Silbersweig DA (2012). Self-awareness, self-regulation, and self-transcendence (S-ART): A framework for understanding the neurobiological mechanisms of mindfulness. Frontiers in Human Neuroscience.

[CR66] de Vignemont F, Singer T (2006). The empathic brain: how, when and why?. Trends in Cognitive Sciences.

[CR67] Walach H, Buchheld N, Buttenmüller V, Kleinknecht N, Schmidt S (2006). Measuring mindfulness-the Freiburg Mindfulness Inventory (FMI). Personality and Individual Differences.

[CR68] Wallace BA (2006). The attention revolution.

[CR69] Woodruff SC, Glass CR, Arnkoff DB, Crowley KJ, Hindman RK, Hirschhorn EW (2014). Comparing self-compassion, mindfulness, and psychological inflexibility as predictors of psychological health. Mindfulness.

