# Supplementary material for: Differential Effects of Attention-, Compassion-, and Socio-Cognitively Based Mental Practices on Self-Reports of Mindfulness and Compassion
Source: Mindfulness (N Y). 2017 Apr 6;8(6):1488–512. doi: 10.1007/s12671-017-0716-z (PMC5693975; doi:10.1007/s12671-017-0716-z)
Supplement: Supplementary file 1 — (DOCX 262 kb) [file 12671_2017_716_MOESM1_ESM.docx]

Supplementary Materials of
Hildebrandt, McCall, & Singer (submitted). Differential Effects of Attention-, Compassion- and Socio-Cognitively Based Mental Practices on Self-Reports of Mindfulness and Compassion.

Additional analyses

In the main paper, we used a slightly uncommon approach for the linear mixed models, because we wanted to account for the longitudinal design, i.e. include an autoregressive correlation structure. In R, this is possible with the lme() function of the nlme package. Unfortunately, this function does not converge if there are empty cells in the design, which is the case with the Test Cohort 3, of which we did not assess data at T2 and T3. In the original mode, we therefore used a single predictor consisting of the combination of test cohort and time point. This approach thus did not allow us to test for main effects. To address this issue, we also conducted more traditional models including two separate predictors (time point and training) and omitting the autoregressive structure using lme4::lmer().

In particular, we fitted three models to each subscale, which we compared using likelihood ratio tests:

A null model: Scores = gender + age_z + random(id);

A reduced model: Scores = gender + age_z + timepoint + random(id);

A main effect model: Scores = gender + age_z + timepoint + training + random(id), and

A full model: Scores = gender + age_z + timepoint * training + random(id).

This allows us to interpret the (main) effect of time points above and beyond the variance explained by the covariates and random effect, as well as the combined effect of training and the interaction between training and time point above and beyond the time point effects. The pattern of results is the same as in the analyses reported in the main manuscript. The definition and results of the contrasts would not change between the two approaches, so the interpretations stay the same.

Table S1

*Results of Likelihood Ratio Tests comparing different nested linear mixed models of the effects of* time *(reduced vs. null) as well as* training *and the interaction between* training *and* time *(full vs reduced) on the different subscales.*

|  | Reduced-null comparison | | | | |  | Main effect-reduced comparison | | | | |  | Interaction-main effect comparison | | | | |
| --- | --- | --- | --- | --- | --- | --- | --- | --- | --- | --- | --- | --- | --- | --- | --- | --- | --- |
|  | χ^2^ |  | df |  | p |  | χ^2^ |  | df |  | p |  | χ^2^ |  | df |  | p |
| FMI |  |  |  |  |  |  |  |  |  |  |  |  |  |  |  |  |  |
| Presence | 143.93 |  | 3 |  | <.001 |  | 28.61 |  | 3 |  | <.001 |  | 4.58 |  | 3 |  | .206 |
| Acceptance | 121.23 |  | 3 |  | <.001 |  | 5.16 |  | 3 |  | .161 |  | 15.08 |  | 3 |  | .002 |
| FFMQ |  |  |  |  |  |  |  |  |  |  |  |  |  |  |  |  |  |
| Observing | 123.69 |  | 3 |  | <.001 |  | 28.99 |  | 3 |  | <.001 |  | 14.45 |  | 3 |  | .002 |
| Describing | 45.79 |  | 3 |  | <.001 |  | 10.63 |  | 3 |  | .014 |  | 5.09 |  | 3 |  | .165 |
| Nonreacting | 96.88 |  | 3 |  | <.001 |  | 28.02 |  | 3 |  | <.001 |  | 5.58 |  | 3 |  | .134 |
| Acting with Awareness | 33.83 |  | 3 |  | <.001 |  | 39.11 |  | 3 |  | <.001 |  | 0.31 |  | 3 |  | .958 |
| Nonjudging | 49.12 |  | 3 |  | <.001 |  | 12.36 |  | 3 |  | 0.006 |  | 1.84 |  | 3 |  | .610 |
| SCS |  |  |  |  |  |  |  |  |  |  |  |  |  |  |  |  |  |
| Self-Kindness | 105.85 |  | 3 |  | <.001 |  | 36.14 |  | 3 |  | <.001 |  | 9.30 |  | 3 |  | .026 |
| Self-Judgment | 58.92 |  | 3 |  | <.001 |  | 7.59 |  | 3 |  | .055 |  | 5.68 |  | 3 |  | .128 |
| Common Humanity | 53.46 |  | 3 |  | <.001 |  | 38.98 |  | 3 |  | <.001 |  | 1. 07 |  | 3 |  | .784 |
| Isolation | 75.53 |  | 3 |  | <.001 |  | 3.33 |  | 3 |  | .344 |  | 4.95 |  | 3 |  | .176 |
| Mindfulness | 79.58 |  | 3 |  | <.001 |  | 44.01 |  | 3 |  | <.001 |  | 1.60 |  | 3 |  | .660 |
| Overidentification | 105.51 |  | 3 |  | <.001 |  | 9.35 |  | 3 |  | .025 |  | 3.99 |  | 3 |  | .262 |
| CS |  |  |  |  |  |  |  |  |  |  |  |  |  |  |  |  |  |
| Kindness | 6.02 |  | 3 |  | .111 |  | 18.51 |  | 3 |  | <.001 |  | 4.40 |  | 3 |  | .221 |
| Indifference | 9.42 |  | 3 |  | .024 |  | 8.07 |  | 3 |  | .045 |  | 0.80 |  | 3 |  | .850 |
| Common | 22.53 |  | 3 |  | <.001 |  | 4.75 |  | 3 |  | .191 |  | 4.00 |  | 3 |  | .262 |
| Separation | 8.81 |  | 3 |  | .032 |  | 6.36 |  | 3 |  | .095 |  | 3.66 |  | 3 |  | .301 |
| Mindfulness | 3.24 |  | 3 |  | .357 |  | 17.87 |  | 3 |  | <.001 |  | 1.88 |  | 3 |  | .598 |
| Disengagement | 9.50 |  | 3 |  | .023 |  | 2.54 |  | 3 |  | .468 |  | 1.41 |  | 3 |  | .703 |
| FoC |  |  |  |  |  |  |  |  |  |  |  |  |  |  |  |  |  |
| Expressing | 95.75 |  | 3 |  | <.001 |  | 28.00 |  | 3 |  | <.001 |  | 9.28 |  | 3 |  | .026 |
| Responding | 41.85 |  | 3 |  | <.001 |  | 3.21 |  | 3 |  | .361 |  | 3.33 |  | 3 |  | .343 |
| Self | 43.23 |  | 3 |  | <.001 |  | 11.98 |  | 3 |  | .008 |  | 5.79 |  | 3 |  | .122 |

*Note.* Null model: Scores = gender + age_z + random(id); Reduced model: Scores = gender + age_z + timepoint + random(id); Main effect model: Scores = gender + age_z + timepoint + training + random(id) ; Interaction model: Scores = gender + age_z + timepoint + training + timepoint * training + random(id).

Table S2

*Results of pairwise comparison contrasts derived from the linear mixed model of the effects of the different trainings and time points on the change scores of the subscales of the* Interpersonal Reactivity Index*.*

|  | *Fantasizing (IRI)* | |  | *Empathic Concern (IRI)* | |  | *Personal Distress (IRI)* | |  | *Perspective Taking (IRI)* | |
| --- | --- | --- | --- | --- | --- | --- | --- | --- | --- | --- | --- |
| Contrast | *Z* | *p* |  | *Z* | *p* |  | *Z* | *p* |  | *Z* | *p* |
| Change between T0 - T1 |  |  |  |  |  |  |  |  |  |  |  |
| Affect - RCC | 1.65 | .099 |  | 3.35 | .001 |  | -2.96 | .003 |  | 5.94 | .000 |
| Pres (both) - RCC | 1.30 | .194 |  | 0.23 | .817 |  | -0.76 | .446 |  | 2.38 | .017 |
| Pres (TC1) - RCC | 1.97 | .049 |  | 1.06 | .287 |  | -0.83 | .406 |  | 2.74 | .006 |
| Pres (TC2) - RCC | 0.24 | .813 |  | -0.69 | .493 |  | -0.47 | .639 |  | 1.32 | .187 |
| Pres (both) - Affect | -0.59 | .554 |  | -3.53 | .000 |  | 2.57 | .010 |  | -4.35 | .000 |
| Pres (TC1) - Affect | 0.34 | .733 |  | -2.17 | .030 |  | 2.02 | .043 |  | -3.02 | .003 |
| Pres (TC2) - Affect | -1.38 | .168 |  | -3.93 | .000 |  | 2.43 | .015 |  | -4.51 | .000 |
| Change between T1 - T2 |  |  |  |  |  |  |  |  |  |  |  |
| Affect - RCC | 0.38 | .705 |  | 1.24 | .215 |  | -0.20 | .840 |  | 2.37 | .018 |
| Persp - RCC | 2.98 | .003 |  | 1.35 | .177 |  | 0.87 | .382 |  | 1.50 | .134 |
| Persp - Affect | 2.53 | .011 |  | 0.10 | .924 |  | 1.05 | .292 |  | -0.88 | .381 |
| Change between T2 - T3 |  |  |  |  |  |  |  |  |  |  |  |
| Affect - RCC | -1.78 | .075 |  | 1.19 | .236 |  | -2.94 | .003 |  | 3.03 | .002 |
| Persp - RCC | 0.65 | .515 |  | 0.76 | .446 |  | -2.70 | .007 |  | 0.84 | .403 |
| Persp - Affect | 2.40 | .016 |  | -0.41 | .681 |  | 0.21 | .836 |  | -2.15 | .031 |
| Change between T1 - T3 |  |  |  |  |  |  |  |  |  |  |  |
| Affect - RCC | -1.09 | .274 |  | 1.92 | .055 |  | -2.52 | .012 |  | 4.22 | .000 |
| Persp - RCC | 2.81 | .005 |  | 1.66 | .096 |  | -1.50 | .135 |  | 1.82 | .069 |
| Persp - Affect | 3.02 | .003 |  | -0.19 | .849 |  | 0.74 | .459 |  | -1.84 | .065 |
| Change between T0 - T3 |  |  |  |  |  |  |  |  |  |  |  |
| Affect - RCC | 0.14 | .886 |  | 3.91 | .000 |  | -4.21 | .000 |  | 7.60 | .000 |

*Note.* Pres = Presence module, Persp = Perspective module, Affect = Affect module, RCC = retest control cohort.


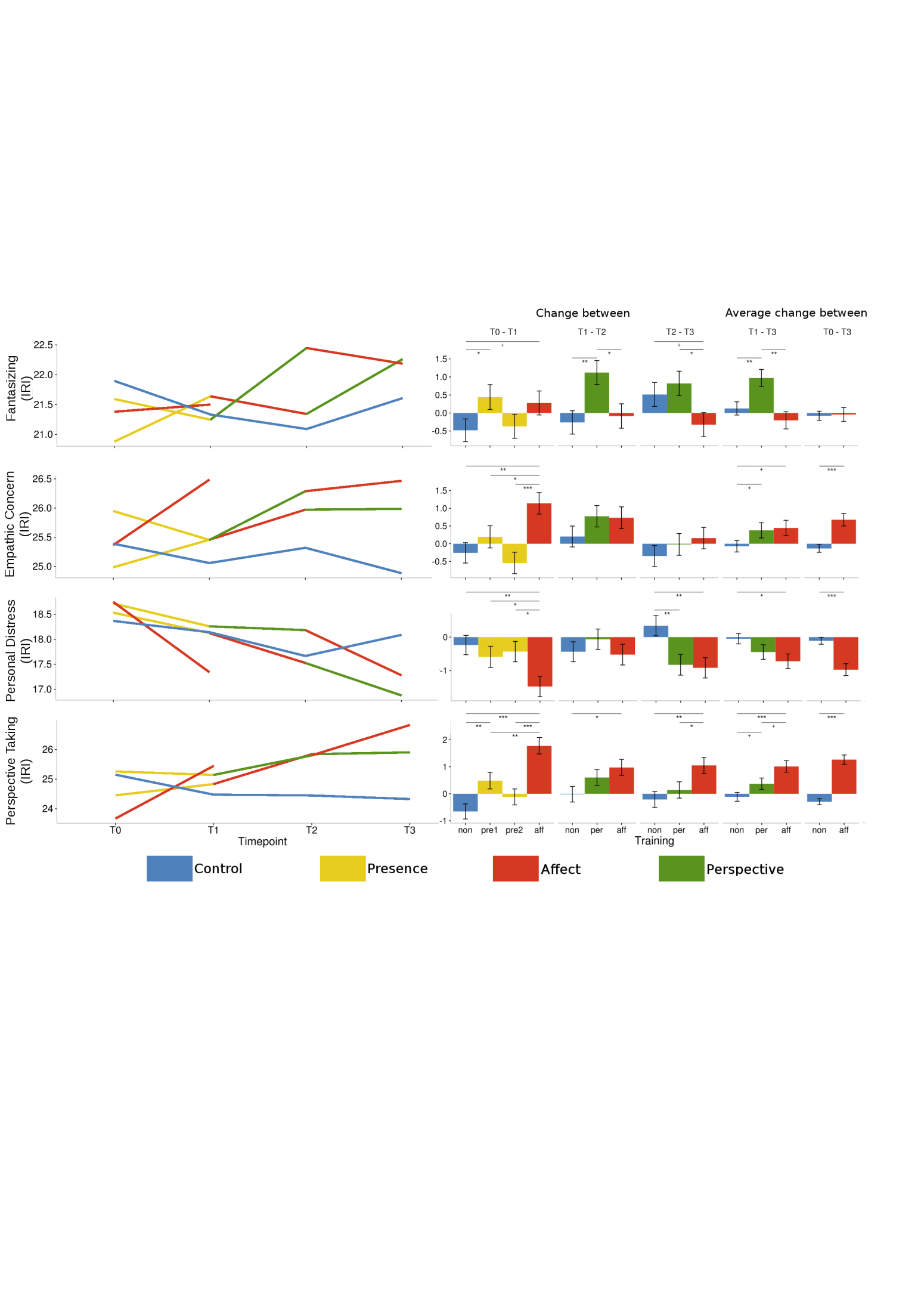


*Figure S1*. Averaged raw data per cohort and time point (left) and estimates and standard errors of the changes between time points derived from the contrasts of the linear mixed models (right) per subscale of the Interpersonal Reactivity Index. The three leftmost bar charts represent the change between two subsequent time points, whereas the two right bar chart columns represent the average change for both Perspective modules (plus the matching control and Affect modules) and the average change for all three Affect modules (plus the matching control cohorts). The full-null model comparisons were all significant. As would have been expected, the contrasts revealed that *fantasizing* scores were increased after the Perspective module, whereas *empathic concern* and *personal distress* ratings were mainly in- or decreased, respectively, by the Affect module, especially at T1. Interestingly, ratings on the *perspective taking* subscale were – contrary to the naming – increased not by the Perspective but mostly by the Affect module, also particularly at T1. This is unexpected as the Perspective module specifically is focused on taking different perspectives and the subscale of the IRI also is aimed at exactly this. *Note*. + *p* <.1; * *p* <= .05; ** *p* <= .01; *** *p* <= .001.
